# Emerging *Aeromonas* enteric infections: their association with inflammatory bowel disease and novel pathogenic mechanisms

**DOI:** 10.1128/spectrum.01088-23

**Published:** 2023-09-21

**Authors:** Seul A. Lee, Fang Liu, Christopher Yuwono, Monique Phan, Sarah Chong, Joanna Biazik, Alfred Chin Yen Tay, Michael Janitz, Stephen M. Riordan, Ruiting Lan, Michael C. Wehrhahn, Li Zhang

**Affiliations:** 1 School of Biotechnology and Biomolecular Sciences, University of New South Wales, Sydney, New South Wales, Australia; 2 Mark Wainwright Analytical Centre, University of New South Wales, Sydney, New South Wales, Australia; 3 Helicobacter Research Laboratory, School of Pathology and Laboratory Medicine, Marshall Centre for Infectious Diseases Research and Training, University of Western Australia, Perth, Australia; 4 Gastrointestinal and Liver Unit, Prince of Wales Hospital, University of New South Wales, Sydney, New South Wales, Australia; 5 Douglass Hanly Moir Pathology, a Sonic Healthcare Australia Pathology Practice, Macquarie Park, New South Wales, Australia; Michigan State University, East Lansing, Michigan, USA

**Keywords:** *Aeromonas*, *Aeromonas veronii*, transcriptome, IBD, intestinal epithelial cells, *Aeromonas caviae*, enteric infection, *Campylobacter*, *Shigella*, *Salmonella*

## Abstract

*Aeromonas* species are emerging human enteric pathogens. This study examines the isolation of *Aeromonas* and other enteric bacterial pathogens from patients with and without inflammatory bowel disease (IBD). This study also investigates the intestinal epithelial pathogenic mechanisms of *Aeromonas veronii*. The isolation rates of seven enteric bacterial pathogens from 2,279 patients with IBD and 373,276 non-IBD patients were compared. An *A. veronii* strain (AS1) isolated from intestinal biopsies of a patient with IBD was used for pathogenic mechanism investigation, and *Escherichia coli* K12 was used as a bacterial control. HT-29 cells were used as a model of human intestinal epithelium. A significantly higher isolation of *Aeromonas* species was found in patients with IBD as compared to non-IBD patients (*P* = 0.0001, odds ratio = 2.11). *A. veronii* upregulated 177 inflammatory genes and downregulated 52 protein-coding genes affecting chromatin assembly, multiple small nuclear RNAs, multiple nucleolar RNAs, and 55 cytoplasmic tRNAs in HT-29 cells. These downregulation effects were unique to *A. veronii* and not observed in HT-29 cells infected with *E. coli* K12. *A. veronii* induced intestinal epithelial apoptosis involving the intrinsic pathway. *A. veronii* caused epithelial microvilli shortening and damage and epithelial production of IL-8. In conclusion, this study for the first time reports the association between IBD and *Aeromonas* enteric infection detected by bacterial cultivation. This study also reports that *A. veronii* damages intestinal epithelial cells via multiple mechanisms, of which the downregulating cytoplasmic tRNA, small nuclear RNA, and small nucleolar RNA are novel bacterial pathogenic mechanisms.

**IMPORTANCE:**

This study for the first time reports the association between inflammatory bowel disease (IBD) and *Aeromonas* enteric infection detected by bacterial pathogen cultivation, highlighting the need of clinical and public health attention. The finding that patients with IBD are more susceptible to *Aeromonas* enteric infection suggests that detection of *Aeromonas* enteric infection should be routinely performed for the diagnosis and treatment of IBD. This study also reports novel bacterial pathogenic mechanisms employed by *Aeromonas veronii*. Through comparative transcriptomic analysis and other techniques, this study revealed the pathogenic mechanisms by which *A. veronii* causes damage to intestinal epithelial cells. Among the various pathogenic mechanisms identified, the downregulating tRNA, small nuclear and nucleolar RNAs in human intestinal epithelial cells are novel bacterial pathogenic mechanisms.

## INTRODUCTION

*Aeromonas* species are facultative anaerobes, gram-negative, rod-shaped bacteria that reside in aquatic environments (1). The *Aeromonas* genus currently encompasses 36 species, several of which are human pathogens causing gastroenteritis and other diseases such as wound infection and bacteremia (1 – 3).

Inflammatory bowel disease (IBD) is a group of chronic inflammatory conditions of the gastrointestinal tract (4). Crohn’s disease (CD) and ulcerative colitis (UC) are the two major forms of IBD (4). The development of IBD is believed to be triggered by various initiating microorganisms such as *Campylobacter* species and enteric pathogens, which lead to the breakdown of the tolerance by the mucosal immune system to gut commensal microbes (5, 6).

Previous studies have examined the clinical presentations and treatment of *Aeromonas* enteric infection in patients with IBD (7 – 10). For example, studies from Lobaton et al. and Guedes et al. reported that patients with IBD infected with *Aeromonas* were treated more often with antibiotics compared to non-IBD patients (7, 8).

In this study, we examined the prevalence of *Aeromonas* enteric infection in patients with IBD in comparison with non-IBD patients. This aspect has not been addressed in previous studies, representing a significant gap in knowledge. We also investigated the pathogenic effects and mechanisms on the intestinal epithelial cells using an *Aeromonas veronii* strain isolated from intestinal biopsies of a patient with IBD via comparative transcriptomic analysis and other experiments. The data from this study provide novel findings regarding *Aeromonas* enteric infection in patients with IBD. We also present novel pathogenic mechanisms employed by *A. veronii* in damaging human intestinal epithelial cells.

## MATERIALS AND METHODS

### Comparison of isolation rates of seven enteric bacterial pathogens from patients with IBD and without IBD

This study utilized data from the isolation of seven enteric bacterial pathogens obtained from 375,842 patients with gastroenteritis symptoms processed at the Douglass Hanly Moir (DHM) Pathology Laboratory Australia between 2015 and 2019. These data were previously analyzed, and the isolation rates of pathogens in different age groups, genders, and seasons were reported (2). In the present study, these data were analyzed to investigate the prevalence of enteric bacterial infections in patients with and without IBD.

For identification of patients with IBD, key words including “IBD,” “inflammatory,” “Crohn,” “ulcerative colitis,” “cd,” and “uc” were used to search the diagnosis recorded for all patients. The obtained initial IBD list was further refined by exclusion of patients with question marks for their IBD diagnosis and IBD family history only. Finally, a total of 375,555 fecal samples, including samples from 2,279 patients with IBD (995 male patients) and 373,276 non-IBD patients (153,295 male patients), were used for comparison of isolations of seven enteric bacterial pathogens including *Aeromonas*, *Campylobacter, Salmonella*, *Shigella*, *Plesiomonas, Vibrio*, and *Yersinia* species.

*Aeromonas* species and other enteric bacterial pathogens analyzed in this study were cultured from fecal samples following the procedures for isolation of enteric pathogens at the DHM, as described previously (2). The same procedures were applied to fecal samples from patients with and without IBD. The presumptive *Aeromonas* isolates were confirmed by oxidase test and matrix assisted laser desorption ionization/time of flight mass spectrometry (MALDI/TOF-MS) (Vitek MS, Biomerieuz, North Ryde, NSW, Australia), and reported as “*Aeromonas* species” due to the possibility of misidentifying *Aeromonas* beyond the genus level by MALDI/TOF-MS.

### Examination of the virulence genes in *Aeromonas veronii* AS1 strain

An *Aeromonas veronii* strain (AS1) isolated at the DHM from intestinal biopsies of a patient with newly diagnosed CD was available to us. The complete genome of *A. veronii* AS1 was sequenced in this study to examine the presence of virulence genes. Briefly, the genome of *A. veronii* AS1 strain was sequenced by Oxford Nanopore and Illumina MiSeq technologies. The details of hybrid assembly and examination of putative virulence factors and secretion systems were described in our previous studies (11, 12). Briefly, for Illumina sequencing, bacterial DNA was extracted using Gentra Puregene Yeast/Bactria Kit (Qiagen), libraries were sequenced on the MiSeq Personal Sequencer, and reads were assembled using Shovill (v 1.0.0). For Nanopore sequencing, bacterial DNA was extracted with phenol-chloroform, libraries were loaded onto FLO-MIN106 flow cell and sequenced on the GridION sequencing device. The complete genome was obtained through hybrid assembly of Illumina and Nanopore reads using Unicycler (v 0.4.7). Putative virulence factors were identified by comparing to known virulence factors in the Virulence Factors Database (13). Secretion systems were examined using MacSyFinder (14).

### Comparative transcriptomic analysis of gene expression responses to *A. veronii* and *Escherichia coli* strain K12 in human intestinal epithelial cells

*A. veronii* strain AS1 was used to examine the pathogenic effects and mechanisms of *A. veronii* infection to human intestinal epithelial cells. The commensal *E. coli* strain K12 was used as a bacterial control. Human intestinal epithelial cell line HT-29 (ATCC no. HTB-38) was used as a model of human intestinal epithelial cells, which were cultured in McCoy’s 5A medium as previously described (15).

HT-29 cells were cultured in six-well plates (2 × 10^6^/well) for 2 days. Cells were incubated with *A. veronii* or *E. coli* K12 in triplicates at multiplicity of infection (MOI) 10 for 4 hours. HT-29 cells without bacterial infection (triplicates) were used as the negative control.

#### Total RNA extraction, library preparation, and RNA sequencing

Total RNA was isolated from the above bacteria-treated and control HT-29 cells using the Isolate II RNA minikit (Bioline). RNA purity and concentration were measured using a NanoDrop spectrophotometer. The RNA integrity was assessed using TapeStation. Samples with RIN score *≥*7 were selected for further analysis.

RNA sequencing (RNA-seq) was conducted at the Ramaciotti Centre for Genomics, University of New South Wales. Briefly, libraries were prepared using Illumina Stranded Total RNA Prep Ligation with Ribo-Zero Plus kit (Illumina) and sequenced on Illumina’s NextSeq 500. At least 60 million 75-bp paired-end reads were generated per sample.

#### Identification of differentially expressed genes

Gene expressions in *A. veronii* and *E. coli* K12-treated HT-29 cells were compared to those in the negative control HT-29 cells. Briefly, sequencing reads were assessed for quality using FastQC (v 0.11.9) and MultiQC (v 0.8). Prior to RNA-seq analysis, Trimmomatic (v 0.40) was used to trim Illumina adapter sequences and filter the raw reads (ILLUMINACLIP, Illumina-PE.fa:2:30:10; LEADING, 3; TRAILING, 3; SLIDINGWINDOW, 4:15; MINLEN, 30) (16). The quality-filtered reads were mapped to the reference human genome GRCh38.p14 using HISAT2 (v 2.1.0). The Subread package Featurecount (v 2.0.1) was used to generate a set of per-gene read counts for each sample using default options (17).

The count data files were loaded into R and analyzed with DESeq2 (v 1.32.0), following standard normalization procedures. Differential gene expressions in HT-29 cells treated with *A. veronii* or *E. coli* compared to non-treated cells were calculated as described in other studies (18, 19). The differentially expressed genes were identified using a *q*-value cut-off of <0.05. Biologically significant differential gene expressions were defined based on *q* < 0.05 and fold change of 2 or more (log_2_ fold change ≥ 1 or log_2_ fold change ≤ −1). The differentially expressed genes were plotted as volcano plots using the EnhancedVolcano package (version 1.14.0) (20).

#### Gene ontology enrichment analysis

The analysis of differentially expressed genes by gene ontology (GO) enrichment was performed using Enrichr, which provides information on how differentially expressed gene sets impact cellular biological processes. The biological processes involved in responses to *A. veronii* or *E. coli* infections in HT-29 cells were identified and ranked by the *P* value (21).

#### Quantitative real-time PCR

To verify the RNA-seq data, five significantly differentially expressed RNAs induced by *A. veronii* and *E. coli* K12 were randomly selected and subjected to quantitative real-time PCR (qRT-PCR), as previously described (15). These were interleukin 1-beta (IL-1β), DNA damage inducible transcript 4 (DDIT4), MIR210HG, chemokine ligand 20 (CCL20), and interleukin-17C (IL17C). The primers used for qRT-PCR are listed in Table S1.

### Staining nucleus and F-actin

HT-29 cells seeded coverslips were incubated for 4 hours with and without *A. veronii*. Cells were then fixed with 3.6% paraformaldehyde, permeabilized with 0.1% Triton X-100, and blocked using 1% bovine serum albumin. F-actin was then stained using Alexa Fluor 488 Phalloidin (Cell Signaling Technology) following the manufacturer’s instructions. The nuclei were stained using Hoechst 33342. Cells were visualized using Olympus fluorescent microscope BX61.

### IL-8 production

The IL-8 production in intestinal epithelial cells induced by *A. veronii* and *E. coli* K12 was measured using enzyme-linked immunosorbent assay kit (Invitrogen). Live and heat-killed *A. veronii* AS1 and *E. coli* K12 were incubated with HT-29 cells at MOI 1 and 10 as previously described (15).

### Caspase 3/7 and caspase 9 activities

HT-29 cells were incubated with *A. veronii* or *E. coli* K12 at MOI 1 and 10 for 2 and 4 hours. Levels of active caspase 3/7 and caspase 9 were measured using CellEvent Caspase-3/7 Green ReadyProbes Reagent (Invitrogen) and CaspGLOW Fluorescein Active Caspase-9 Staining Kit (Invitrogen) following the manufacturer’s instructions.

### Examining the interaction between *A. veronii* and intestinal epithelial cells

#### Gentamicin protection assay

HT-29 cells were infected with *A. veronii* AS1, *E. coli* K12 (negative control), *E. coli* L20 (positive control for adhesion), and *Salmonella enterica* serovar Typhimurium (positive control for invasion) for 4 hours. Gentamicin protection assay was performed as previously described to examine the adhesion and invasion of *A. veronii* to HT-29 cells (15).

#### Scanning and transmission electron microscopy

Scanning electron microscopy (SEM) and transmission electron microscopy (TEM) were used to further investigate the adhesive and invasive abilities of *A. veronii* AS1, as well as to visualize morphological changes in HT-29 cells caused by the bacterium. HT-29 cells were infected with *A. veronii* AS1 at MOI 10 for 2 and 4 hours. SEM and TEM analyses were performed at Mark Wainwright Analytical Centre, Electron Microscope Unit, University of New South Wales.

Briefly, samples were fixed overnight at 4°C using a fixative containing glutaraldehyde in 0.2 M sodium phosphate buffer. Fixed samples were then washed with 0.1 M sodium phosphate buffer followed by dehydration using ethanol. Next, samples were dehydrated using increasing concentrations of hexamethyldisilizane (HMDS) and left to air-dry in a 100% solution of HMDS. Samples were mounted onto SEM stubs, platinum coated, and viewed using an FEI Nova NanoSEM 230 (Oregon) operating at 5 kV.

For TEM, fixed samples were rinsed with 0.1 M sodium phosphate buffer and post-fixed in 1% osmium tetroxide in 0.2 M sodium buffer by using a BioWave Pro+ Microwave Tissue Processor (Ted Pella). After rinsing with 0.1 M sodium phosphate buffer, samples were dehydrated in a graded series of ethanol, infiltrated with resin (Procure, 812), and polymerized using an oven at 60°C for 48 hours. Ultrathin sections (70 nm) were cut using a diamond knife (Diatome) and collected onto carbon-coated copper slot TEM grids. Grids were post-stained using uranyl acetate (2%) and lead citrate. Samples were imaged using a JEOL 1400 TEM operating at 100 kV.

### Statistical analysis

The isolation rates of enteric bacterial pathogens in patients with and without IBD were compared by a logistic regression analysis using a binomial generalized linear model in the R Statistical Package (version 4.0.1), with IBD as a categorical independent variable, and the ratio of positive isolation case numbers to the total fecal samples in each patient group, sex, and age as the dependent variables.

For differential gene expression analysis, Wald tests embedded in the DESeq2 package were applied. A one-way analysis of variance (ANOVA) with Dunnett’s test was performed to compare the mRNA levels determined by qRT-PCR, IL-8 concentrations, and caspase activities between samples. *P* < 0.05 was considered statistically significant.

## RESULTS

### *Aeromonas* enteric infection is positively associated with IBD

The isolation rate of *Aeromonas* species from patients with IBD was 1.18% (27/2279), which was significantly higher than that from non-IBD patients (0.56%, 2105/373,276), with *P* = 0.0001, the odds ratio (OR) being 2.11 after adjusting for sex (age had no effects in comparison), and 95% confidence interval (CI) of 1.44 to 3.09. The isolation rates of *Campylobacter* and *Salmonella* species in IBD patients were significantly lower than those in non-IBD patients (Table 1). The isolation rates of *Shigella*, *Vibrio*, and *Yersinia* species in patients with and without IBD were not statistically different (Table 1).

**TABLE 1:** The associations between IBD and enteric infections of *Aeromonas* species and other six enteric bacterial pathogens^*a*^

	*Aeromonas*	*Campylobacter*	*Salmonella*	*Plesiomonas*	*Shigella*	*Vibrio*	*Yersinia*
IBD	1.18% (27)	1.32% (30)	0.75% (17)	0.13% (3)	0	0.09% (2)	0.04% (1)
Non-IBD	0.56% (2,105)	3.10% (11,553)	1.39% (5,185)	0.06% (225)	0.07% (258)	0.03% (106)	0.02% (70)
*P* value	0.0001	<0.0001	0.009	0.18	0.908	0.119	0.406
Odds ratio (95% CI)^*b*^	2.11 (1.44–3.09)	0.412 (0.287–0.591)	0.53 (0.328–0.855)	NA^*c*^	NA	NA	NA

^*a*^ A total of 375,555 fecal samples from patients with gastroenteritis symptoms subjected to enteric bacterial pathogen isolation were included for analysis, including samples from 2,279 patients with IBD (average age ± SD: 40.47 ± 19.89, 995 male patients) and 373,276 non-IBD patients (average age ± SD: 40.6 ± 27.55, 153,295 male patients). The percentages for each enteric bacterial pathogen were the positive isolation case numbers (numbers in brackets) divided by fecal samples of each patient group. The isolation rates of enteric bacterial pathogens in patients with and without IBD were compared by a logistic regression analysis using a binomial generalized linear model in the R statistical package (version 4.0.1), with IBD as a categorical independent variable, and the ration of positive isolation case numbers to the total fecal samples in each patient group, sex, and age as the dependent variables. *P* < 0.05 indicates a statistically significant result.

^*b*^ CI, confidence interval.

^*c*^ NA, not applicable.

### *A. veronii* induced novel gene expression patterns in human intestinal epithelial cells in comparison to *E. coli* K12

**Fig 1:**
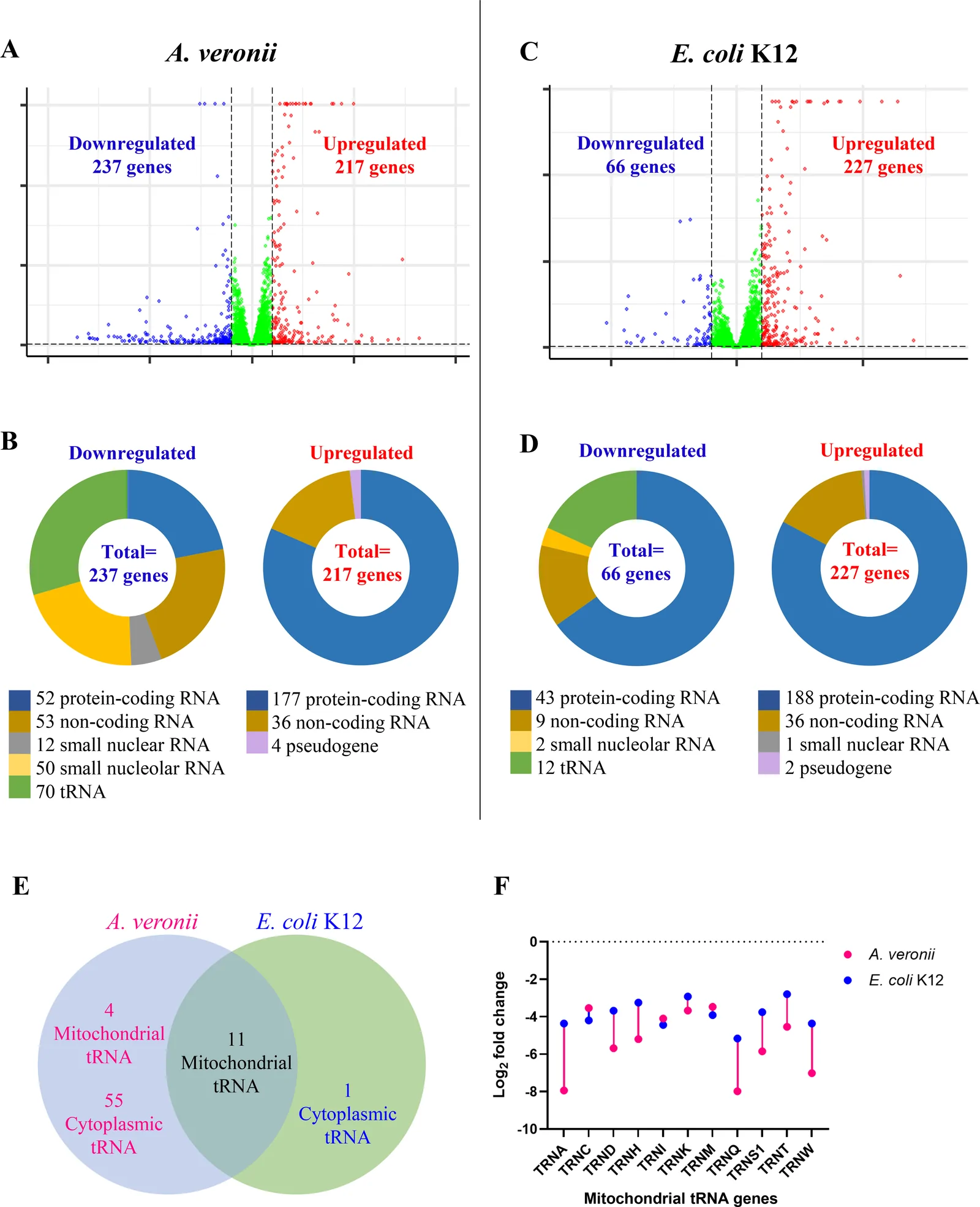
*A. veronii* and *E. coli* K12 upregulated and downregulated expression of genes in human intestinal epithelial HT-29 cells. (A and B) Volcano plot of differentially expressed genes following incubation with *A. veronii* and *E. coli*, respectively, for 4 hours as compared with the untreated control cells. The cut-off log_2_ fold change was ≥1 and ≤ −1, and adjusted *P* value was <0.05. (B and D) Composition of genes upregulated by *A. veronii* and *E. coli*, respectively. (E) Comparison of tRNAs downregulated by *A. veronii* and *E. coli*. (F) Fold changes of the 11 mitochondrial tRNAs commonly downregulated by both *A. veronii* and *E. coli*. Lines between the data points were colored based on the bacterium that induced the more fold changes. All 11 tRNAs have logarithmic fold change <1 and *q* < 0.05.

Transcriptomic analysis revealed that both *A. veronii* and *E. coli* K12 significantly altered the expression of 454 and 293 genes in HT-29 cells (*q* < 0.05 and twofold or more changes), respectively. *A. veronii* infection resulted in downregulation of more transcripts than *E. coli* K12-infected cells (237 vs 66, respectively) (Fig. 1A and C).

The upregulated transcripts by both *A. veronii* and *E. coli* K12 (217 and 227, respectively) were mainly expressed by protein-coding genes involved in inflammation (Fig. 1B and D; Table S2). *A. veronii* downregulated 52 protein-coding RNAs, 10 of which encoded histone proteins. *E. coli* downregulated 43 protein-coding RNAs, eight of which coded for heat shock proteins.

*A. veronii* downregulated 12 small nuclear RNAs, none of which were affected by *E. coli* K12. *A. veronii* downregulated 50 small nucleolar RNAs, of which 48 were specific to *A. veronii* and two were also downregulated by *E. coli* K12 (Fig. 1B and D; Table S2).

Interestingly, *A. veronii* downregulated 70 tRNAs, including 55 cytoplasmic tRNAs and 15 mitochondrial tRNAs (Fig. 1E). Eleven of these 15 mitochondrial RNAs were downregulated by both *A. veronii* and *E. coli* K12; however, the levels downregulated by *E. coli* K12 were usually lower (Fig. 1F).

The expression levels of five randomly selected genes, including IL-1β, DDIT4, MIR210HG, CCL20, and IL17C, in HT-29 cells infected with *A. veronii* or *E. coli*, and the control HT-29 cells were also tested using qRT-PCR, which showed consistent results with expression patterns revealed using RNA-seq (Fig. S1).

### *A. veronii* downregulated chromatin and nucleosome assembly

**Fig 2:**
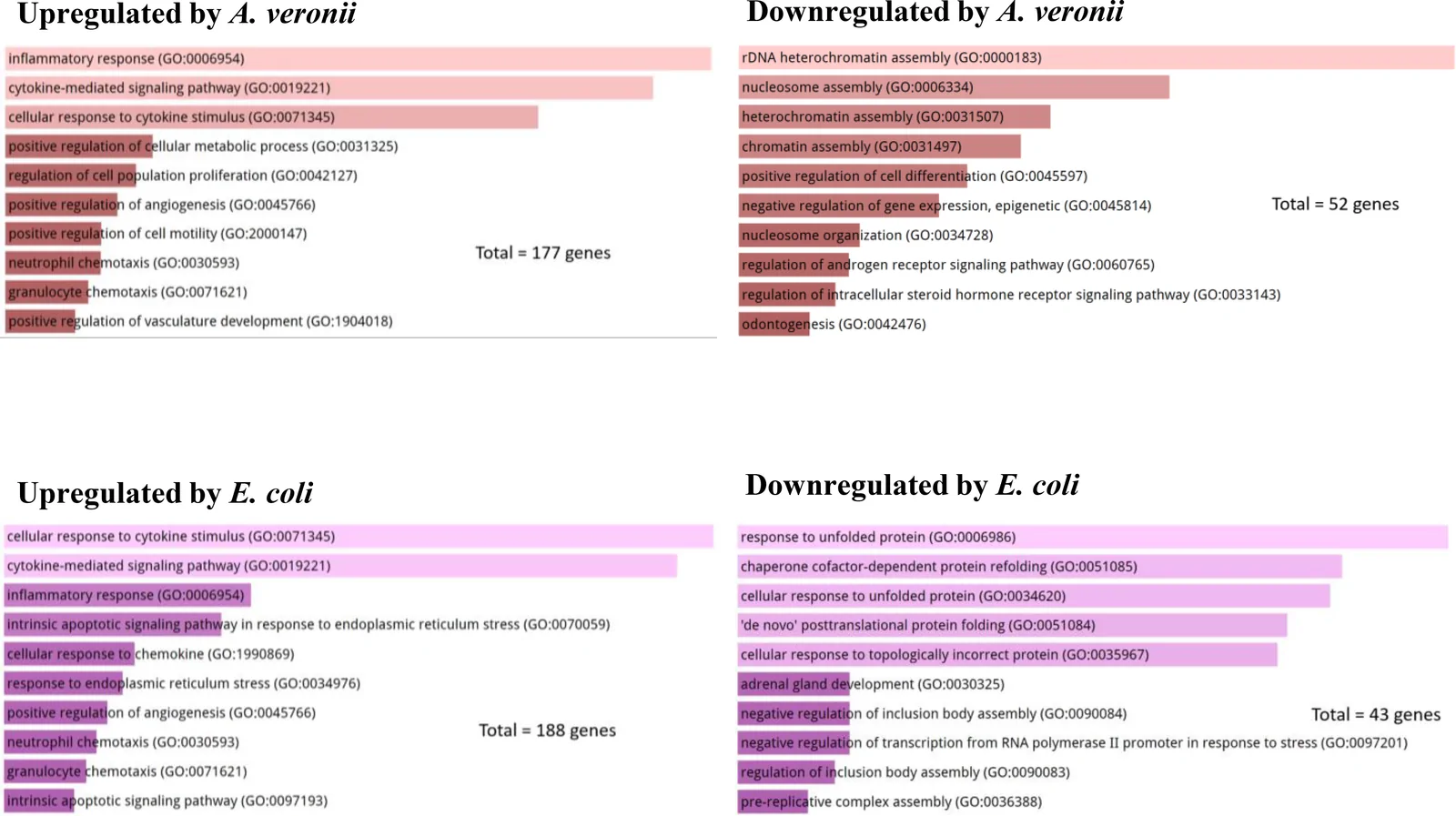
The impact of *A. veronii* and *E. coli* K12 on biological processes in human intestinal epithelial HT-29 cells. The top 10 enriched gene ontology (GO) terms of protein-coding genes in biological process group are presented, ranked based on *P* values from lowest to highest. Both *A. veronii* AS1 and *E. coli* K12 upregulated biological processes associated with inflammation in HT-29 cells. However, *A. veronii* AS1 specifically downregulated the top four biological processes related to chromatin assembly, while *E. coli* K12 downregulated the top five biological processes associated with responses to unfolded proteins in HT-29 cells.

The top 10 (ranked by *P* value) significantly enriched GO terms in biological process group are displayed in Fig. 2. The upregulated biological processes by *A. veronii* and *E. coli* infection were mainly related to inflammation such as inflammatory response and cytokine-mediated signaling pathway (Fig. 2). The striking differences between *A. veronii* and *E. coli* in biological impact on cellular physiology in HT-29 cells were from the enrichment of downregulated genes. *A. veronii* affected chromatin and nucleosome assembly, while *E. coli* mainly affected protein refolding (Fig. 2).

### *A. veronii* damaged the chromatin structure

**Fig 3:**
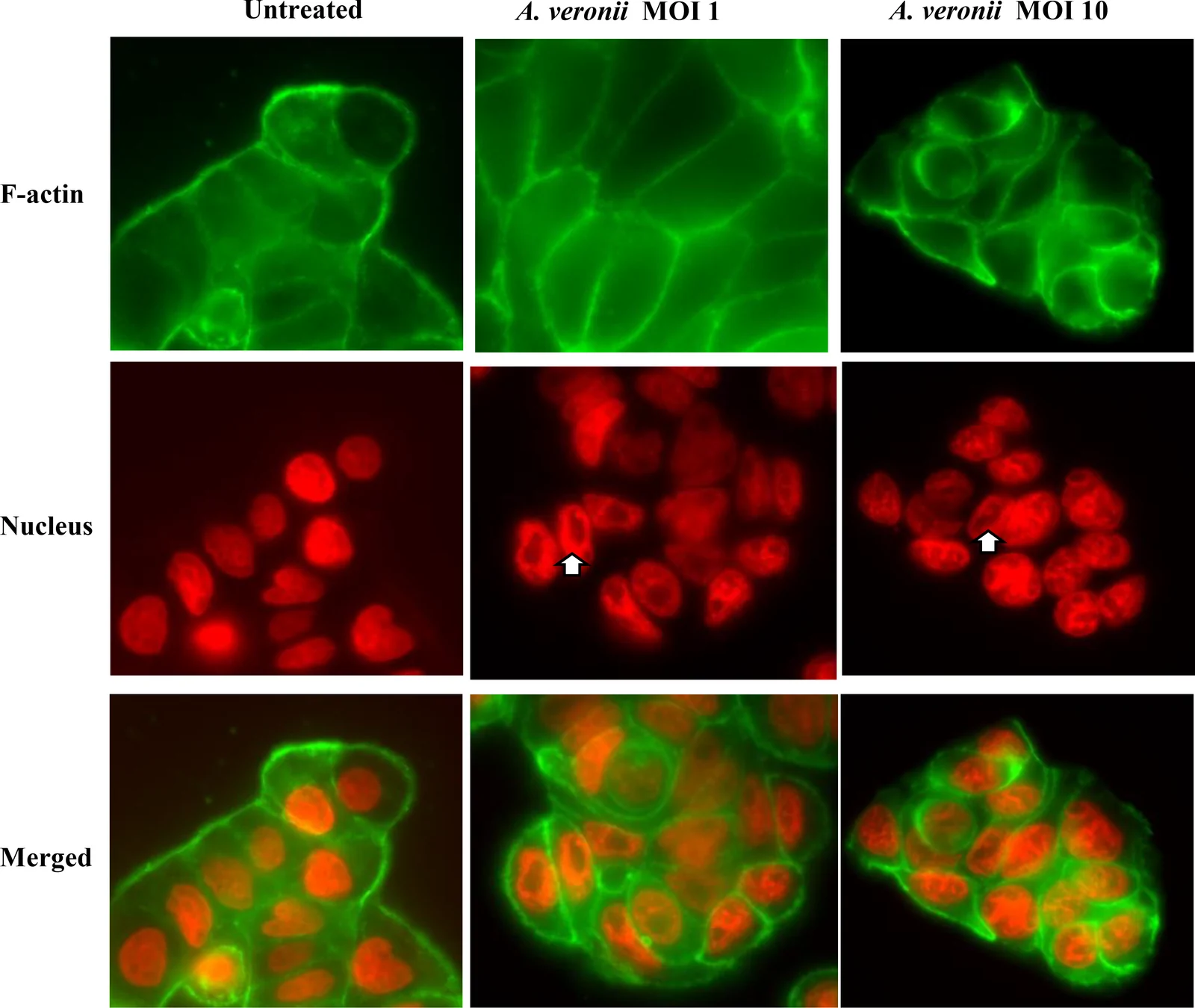
*A. veronii* caused aberrant chromatin arrangement in human intestinal epithelial HT-29 cells. The fluorescent staining of F-actin and nuclei of HT-29 cells is presented. Compared to HT-29 cells without bacterial infection (untreated), HT-29 cells infected with *A. veronii* for 4 hours at both MOI 1 and MOI 10 showed an aberrant chromatin arrangement, as indicated by the hollow unstained areas in cell nuclei.

Nuclear staining of HT-29 cells showed the adverse effect of *A. veronii* on epithelial cell chromatin structure. In HT-29 cells infected with *A. veronii*, the chromatins displayed hollow unstained areas which were not observed in the control HT-29 cells (Fig. 3).

### IL-8

**Fig 4:**
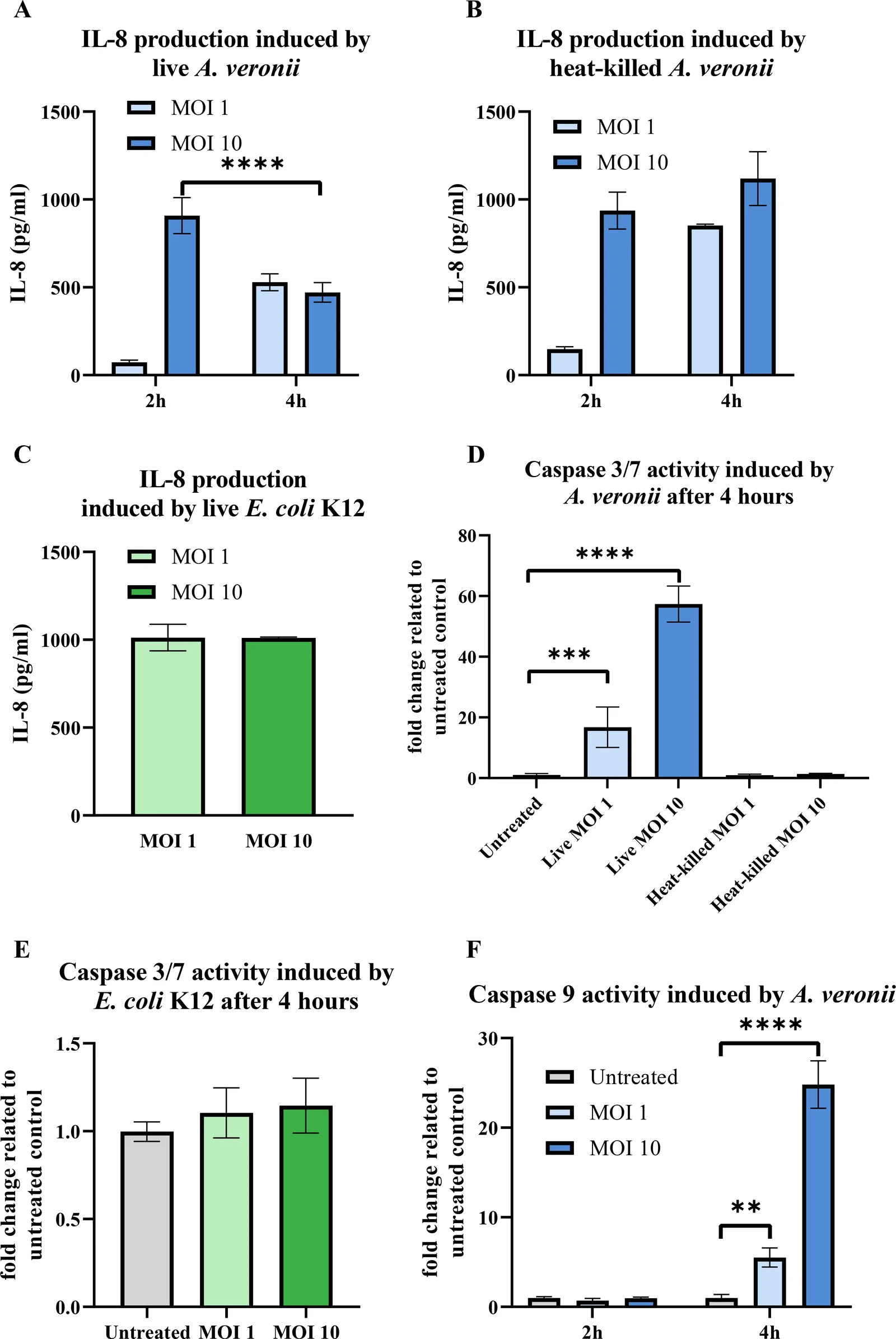
*A. veronii* induced proinflammatory cytokine production, caspase 3/7, and caspase 9 activities in human intestinal epithelial HT-29 cells. (A) Live *A. veronii* bacteria induced IL-8 production. (B) Heat-killed *A. veronii* bacteria also induced IL-8 production. The levels of IL-8 are presented after subtraction of IL-8 levels of the untreated control cells. (C) Live *E. coli* bacteria induced IL-8 production. (D) Live *A. veronii* bacteria induced significantly higher levels of caspase 3/7 activity. (E) Live *E. coli* bacteria did not increase caspase 3/7 activity. (F) *A. veronii* bacteria significantly increased the level of caspase 9 following 4 hours of incubation. A one-way analysis of variance with Dunnett’s test was performed for statistical analysis. Asterisk (*) indicates statistical significance (*P < 0.05, **P < 0.01, and ****P < 0.0001).

Both *A. veronii* and *E. coli* K12 induced IL-8 production in HT-29 cells. Following 2-hour incubation, the live *A. veronii* bacteria and heat-killed *A. veronii* bacteria at MOI 10 induced a similar level of IL-8 production (908 ± 102 and 937 ± 105 pg/mL, respectively). However, following 4-hour incubation, live *A. veronii* bacteria at MOI 10 induced IL-8 to be significantly decreased (529 ± 48 and 471 ± 56 pg/mL, respectively) as compared to the IL-8 level of the 2-hour incubation and the heat-killed *A. veronii* (Fig. 4A and B). Following 4-hour incubation, live *E. coli* bacteria at MOI 1 and 10 induced IL-8 production to 1,012 ± 76 and 1,010 ± 5 pg/mL, respectively (Fig. 4C).

### *A. veronii* induced intestinal epithelial cell apoptosis via the intrinsic pathway

*A. veronii* at both MOI 1 and MOI 10 induced a significantly higher level of caspase 3/7 in HT-29 cells as compared to non-treated HT-29 control cells (*P* < 0.0001), with fold changes of 16.78 ± 3.32 and 57.39 ± 2.66, respectively (Fig. 4D). *A. veronii* also induced increased caspase 9 activities (Fig. 4F), indicating the involvement of the intrinsic pathway in *A. veronii* induced apoptosis. Heat-killed *A. veronii* bacteria did not induce an increase of caspase 3/7 in HT-29 cells compared to non-treated control HT-29 cells (Fig. 4C). Similarly, live *E. coli* K12 bacteria did not increase caspase 3/7 in HT-29 cells (Fig. 4E).

### *A. veronii* adhered to intestinal epithelial cells and caused microvilli shortening

**Fig 5:**
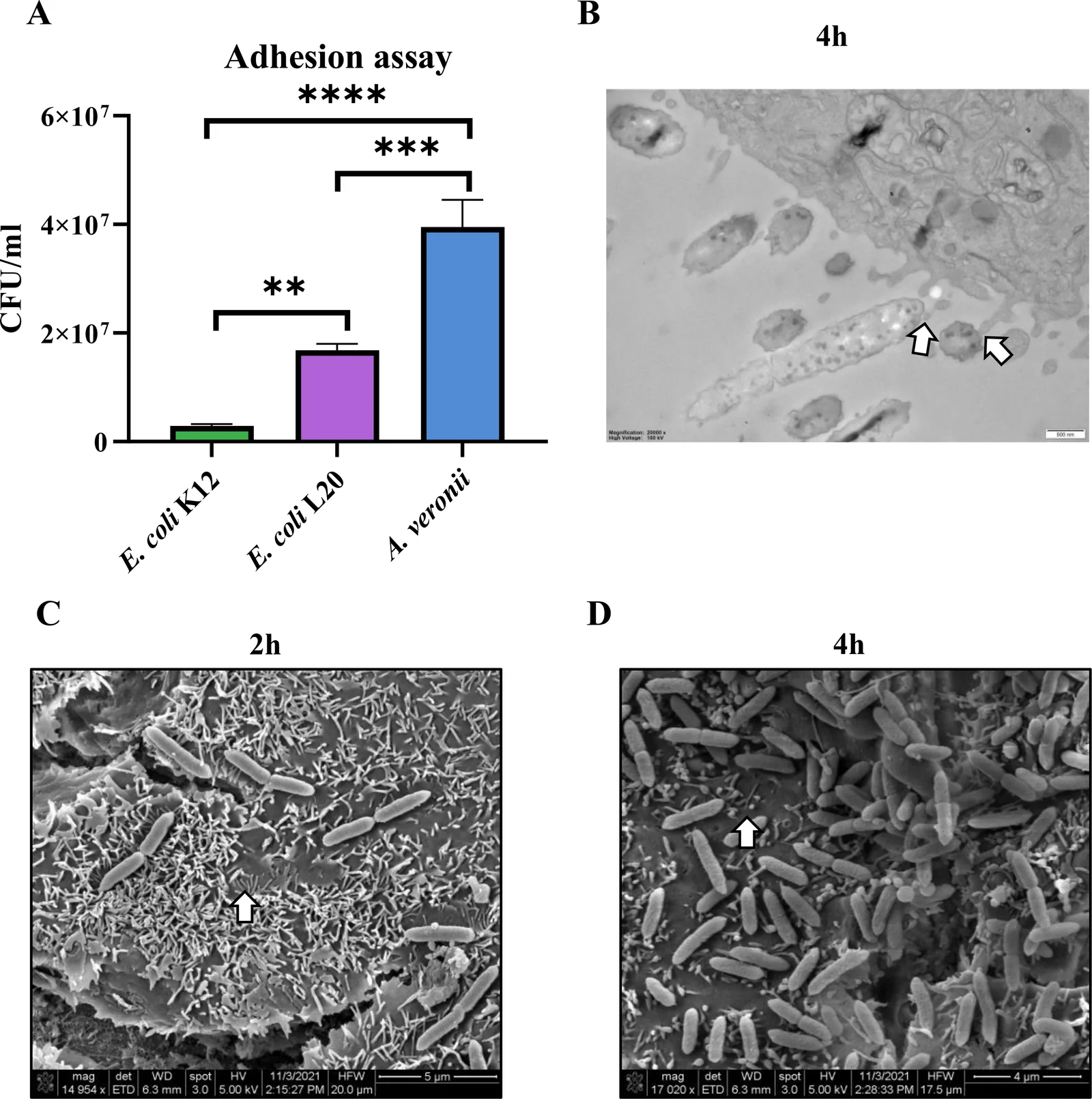
Adhesion of *A. veronii* to human intestinal epithelial HT-29 cells. (A) Both *A. veronii* AS1 and the positive control *E. coli* L20 showed a significantly higher level of adhesion to HT-29 cells as compared to that of the negative control *E. coli* K12 after 4 hours of incubation. (B) TEM shows the attachment of *A. veronii* to HT-29 cells after 4 hours of incubation. (C and D) SEM shows *A. veronii* caused microvilli shortening or disappearance, as indicated by white arrows. A one-way analysis of variance with Dunnett’s test was performed for statistical analysis. Asterisk (*) indicates statistical significance (**P < 0.01, ***P < 0.001, and ****P < 0.0001).

*A. veronii* showed significantly higher levels of adhesion compared to *E. coli* K12 and *E. coli* L20 (*P* < 0.05). Values for adhesion for HT-29 cells infected with *A. veronii*, *E. coli* K12, and *E. coli* L20 were 3.95E7 ± 5.00E6 CFU/mL, 2.88E6 ± 3.43E5 CFU/mL, and 1.68E07 ± 1.17E5 CFU/mL, respectively (Fig. 5A). No *A. veronii* growth from lysed HT-29 cells after gentamicin treatment was observed, showing no bacterial invasion to HT-29 cells. SEM and TEM both showed adhesion of *A. veronii* to HT-29 cells (Fig. 5B, C, and D). SEM revealed that *A. veronii* caused the shortening or disappearance of microvilli, which was observed to initiate at 2 hours and became more pronounced after 4 hours (Fig. 5C and D), and TEM showed membrane blebbing in *A. veronii*-treated cells (Fig. 5D).

### *A. veronii* strain AS1 genome and virulence factors

**Fig 6:**
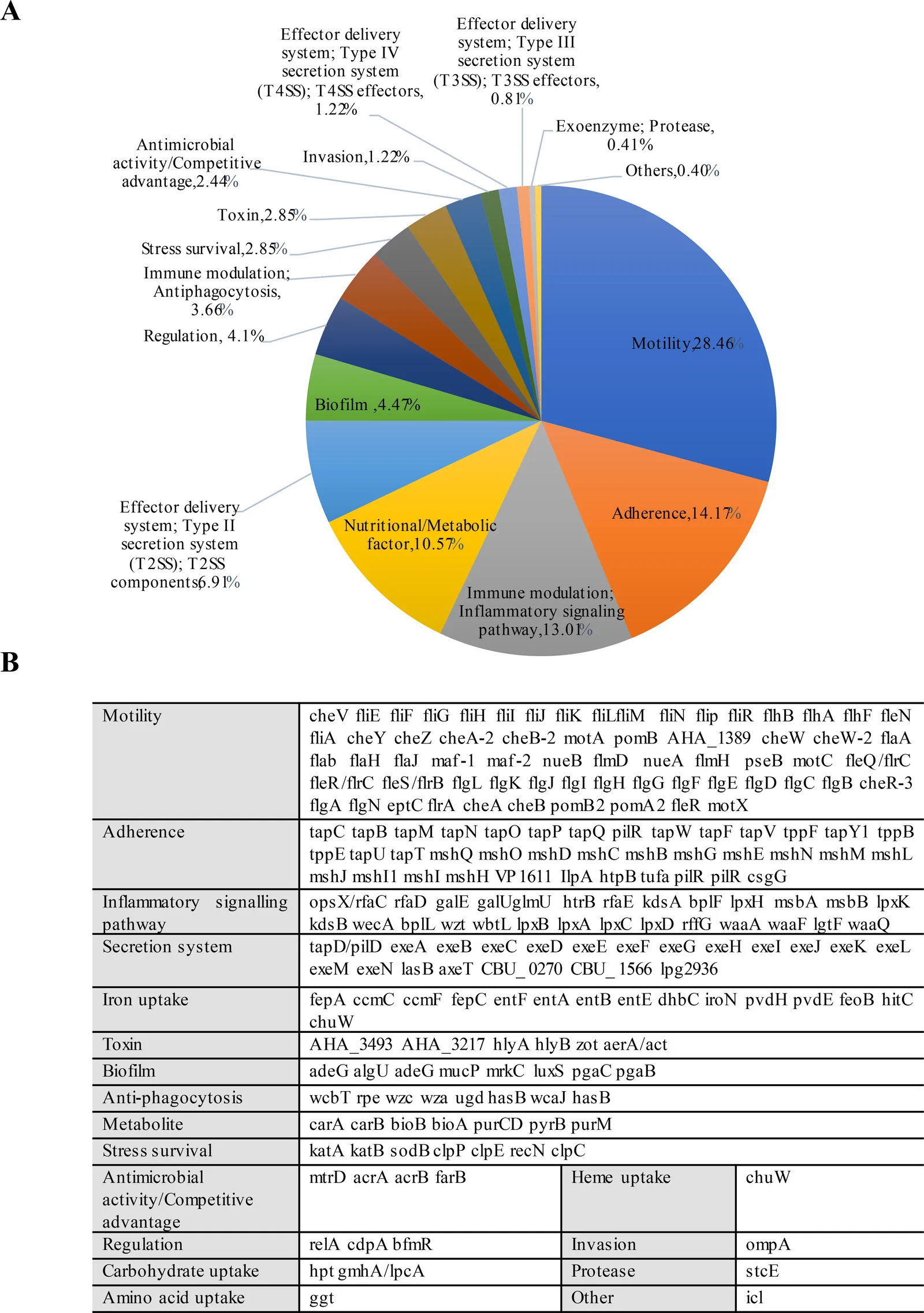
Putative virulence factors in *Aeromonas veronii* strain AS1. *A. veronii* strain AS1 was isolated from intestinal biopsies of a patient with newly diagnosed Crohn’s disease. The complete genome of *A. veronii* strain AS1 was sequenced in this study, and putative virulence factors were identified through searches of the Virulence Factors Database. A total of 246 virulence genes were identified in *A. veronii* AS1 genome. Genes encoding secreted toxins such as aerolysin, hemolysins, microbial collagenase, and StcE (secreted protease of C1-esterase inhibitor) were identified. T1SS and T2SS secretion systems were found in the genome of *A. veronii* strain AS1. However, T3SS, T4SS, and T6SS secretion systems were found to be absent.

The complete genome of *A. veronii* strain AS1 was successfully sequenced. Two hundred and forty-six putative virulence genes were identified in *A. veronii* AS1. Genes encoding secreted toxins such as aerolysin, hemolysins, microbial collagenase, and StcE (secreted protease of C1-esterase inhibitor) were present (Fig. 6). T1SS and T2SS were found in the genome of *A. veronii* strain AS1. T3SS, T4SS, and T6SS were absent.

## DISCUSSION

*Aeromonas* species are emerging human enteric pathogens. However, much remains unknown about the prevalence of *Aeromonas* enteric infection in patients with IBD and the *Aeromonas* enteric pathogenic mechanisms. In this study, we investigated the enteric infection rates of *Aeromonas* and other six enteric bacterial pathogens in patients with and without IBD and examined the intestinal epithelial pathogenic effects and mechanisms induced by *A. veronii* via comparative transcriptomic analysis. Our study discovered an association between the prevalence of *Aeromonas* enteric infection and IBD, as well as novel bacterial pathogenic mechanisms.

Through comparison of the bacterial culture positivity of *Aeromonas* and other six enteric pathogens in patients with and without IBD who presented with gastroenteritis symptoms, we found a positive association between IBD and enteric infection of *Aeromonas* species detected by bacterial culture from fecal samples (OR = 2.11), showing that patients with IBD are more susceptible to *Aeromonas* enteric infections (Table 1). We also found a significantly lower infection rate of *Campylobacter* and *Salmonella* in patients with IBD as compared to non-IBD patients. However, the reason why patients with IBD had a higher *Aeromonas* enteric infection, but a lower *Campylobacter* and *Salmonella* infection is not clear.

Our study did not have the capacity to determine whether *Aeromonas* enteric infection plays a role in the pathogenesis of IBD. However, previous studies suggest that *Aeromonas* enteric infection may play a role in triggering the development of IBD or relapse in some patients (7, 9, 10). For example, Lobaton et al. conducted a study in Belgium, involving 11 IBD patients and 66 non-IBD patients, and found that *Aeromonas* enteric infection triggered a moderate to severe flare in two patients with UC (7). They also reported the isolation of *Aeromonas* species from two patients with newly diagnosed CD (4). Another study by Willoughby et al. from the UK reported that three patients progressed to UC after *Aeromonas hydrophila* or *Aeromonas sobria* infection (9). Despite the interesting findings, these earlier studies had limited sample sizes. Therefore, it is crucial to conduct future longitudinal prospective studies involving larger cohorts of patients to investigate the potential role of *Aeromonas* enteric infection in the pathogenesis of IBD.

Currently, the routine screening for enteric bacterial enteric pathogens in many diagnostic laboratories does not include *Aeromonas* species. Our finding that patients with IBD are more prone to *Aeromonas* enteric infection highlights the importance of screening for *Aeromonas* species in patients with IBD presenting with gastrointestinal symptoms. By implementing such screening measures, targeted antibiotic intervention can be promptly provided. This viewpoint is further supported by previous clinical studies, which showed that patients with IBD infected with *Aeromonas* experienced more complications and required more frequent antibiotic treatment as compared to non-IBD patients (7, 8).

Several *Aeromonas* species such as *A. veronii*, *Aeromonas caviae*, and *A. hydrophila* have been isolated from patients with and without IBD (1 – 4). Lobaton et al. reported that *A. veronii* more frequently caused severe infection than the other species (7). In the DHM diagnostic laboratory, *Aeromonas* isolates were reported as “*Aeromonas* species” due to the potential for misidentification beyond the genus level by MALDI/TOF-MS. In our study, we had access to an *Aeromonas* isolate cultured from intestinal biopsies of a patient with newly diagnosed CD. The complete genome sequencing of this isolate revealed it to be *A. veronii* (AS1 strain), which was further used for transcriptomic analyses and examination of virulence genes. We identified over 200 virulence genes within the genome of *A. veronii* AS1 strain (Fig. 6). Some of these virulence factors have been characterized in other bacteria. For example, aerolysin in *A. hydrophila* acts as a pore-forming toxin (22, 23). StcE metalloprotease was initially found in enterohaemorrhagic *E. coli* O157:H7, which cleaves C1 inhibitor and mucins (24). In a previous study, we examined the genomes of 168 *A*. *veronii* strains isolated from various sources, including patients with gastroenteritis, and found that 106 *A*. *veronii* strains (63%) possessed T3SS secretion systems (7). T3SS secretion systems enable bacterial pathogens to inject bacterial effector proteins directly into host cell cytoplasm (25). In our previous study, we found that 48% *A*. *veronii* strains isolated from patients with gastrointestinal infections possessed T3SS system (7). However, the AS1 strain sequenced in this study lacks the T3SS system. Future studies should aim to examine the genomes of *Aeromonas* strains isolated from patients with IBD and other human diseases to identify genomic features that may be potentially associated with different diseases.

We employed comparative transcriptomic analysis to identify the biological pathways that are disturbed in human intestinal epithelial cells by *A. veronii*, a leading *Aeromonas* enteric pathogen. *E. coli* K12 strain, a commensal gut bacterium with some properties common to *A. veronii* such as being a facultative anaerobic motile rod, was used as the control bacterial species. RNAs upregulated by *A. veronii* were mainly from protein-coding genes related to inflammation such as CCL20, IL-8, and IL-17C. Many of these molecules were also upregulated by *E. coli* K12, showing that they were the common epithelial responses to bacteria (Fig. 1; Table S2). However, IL-1β RNA was upregulated only by *A. veronii*, suggesting that some specific responses were triggered toward *A. veronii*.

The RNAs downregulated by *A. veronii* and *E. coli* K12 presented a markedly different picture. Nearly 20% of protein-coding RNAs downregulated by *A. veronii* were expressed by genes coding for histones, which were not seen in *E. coli* K12 treated HT-29 cells (Table S2). The histone proteins are for packaging DNA into nucleosomes, then condensing them into chromatin (26, 27). The biological process of nucleosome and chromatin assemblies in intestinal epithelial cells was clearly affected by *A. veronii* (Fig. 2). When the nuclei of intestinal epithelial HT-29 cells were stained, it was revealed that the chromatin structure of HT-29 cells was indeed damaged by *A. veronii* (Fig. 3). Chromatin is essential for fundamental cell processes such as DNA replication, transcription, and cell division (28). *A. veronii* also specifically downregulated 12 small nuclear RNAs and 48 nucleolar RNAs. These RNAs are for splicing of introns from pre-mRNA and maturation of ribosomal RNAs, respectively (29 – 31). *A. veronii* also downregulated large numbers of tRNAs which are critical for protein synthesis (32, 33). Overall, *A. veronii* downregulated the cellular processes of intestinal epithelial cells at both transcriptional and translational levels. One direct consequence is that such a downregulation may adversely affect the production of epithelial cytokines, resulting in compromised immune responses. Indeed, we showed that live *A. veronii* bacteria compromised the production of IL-8 as compared to dead *A. veronii* bacteria and live *E. coli* K12 (Fig. 4).

tRNAs are also known to inhibit apoptosis by blocking the activation of caspase 9 through binding to cytochrome c, which impairs the formation of apoptosome (34). Degradation of tRNA using RNA hydrolysis was previously demonstrated to enhance apoptosis via the intrinsic pathway (34). *A. veronii* induced apoptosis in HT-29 cells as indicated by the increased caspase 3/7 activities, and the apoptosis was involved in the intrinsic pathway as indicated by increased caspase 9 activities (Fig. 4). These data strongly support the role of tRNA downregulation by *A. veronii* in contributing to intestinal epithelial cell apoptosis. *A veronii* at MOI 10 induced a higher level of caspase 3/7 in HT-29 cells than MOI 1 (Fig. 4), suggesting that patients infected with a higher load of *A. veronii* may develop more severe clinical symptoms. Heat-killed *A. veronii* induced IL-8 production in HT-29 cells but not apoptosis, showing that the virulence factors responsible for epithelial cell death were heat labile (Fig. 4).

*A. veronii* adhered to intestinal epithelial cells, reduced epithelial microvilli, and caused membrane blebbing (Fig. 5). Previous studies have shown that reorganization of cytoskeletal structures drives the formation of membrane blebs and apoptotic bodies and accompanying the formation of membrane blebbing is the loss of cell surface microvilli (35 – 38).

**Fig 7:**
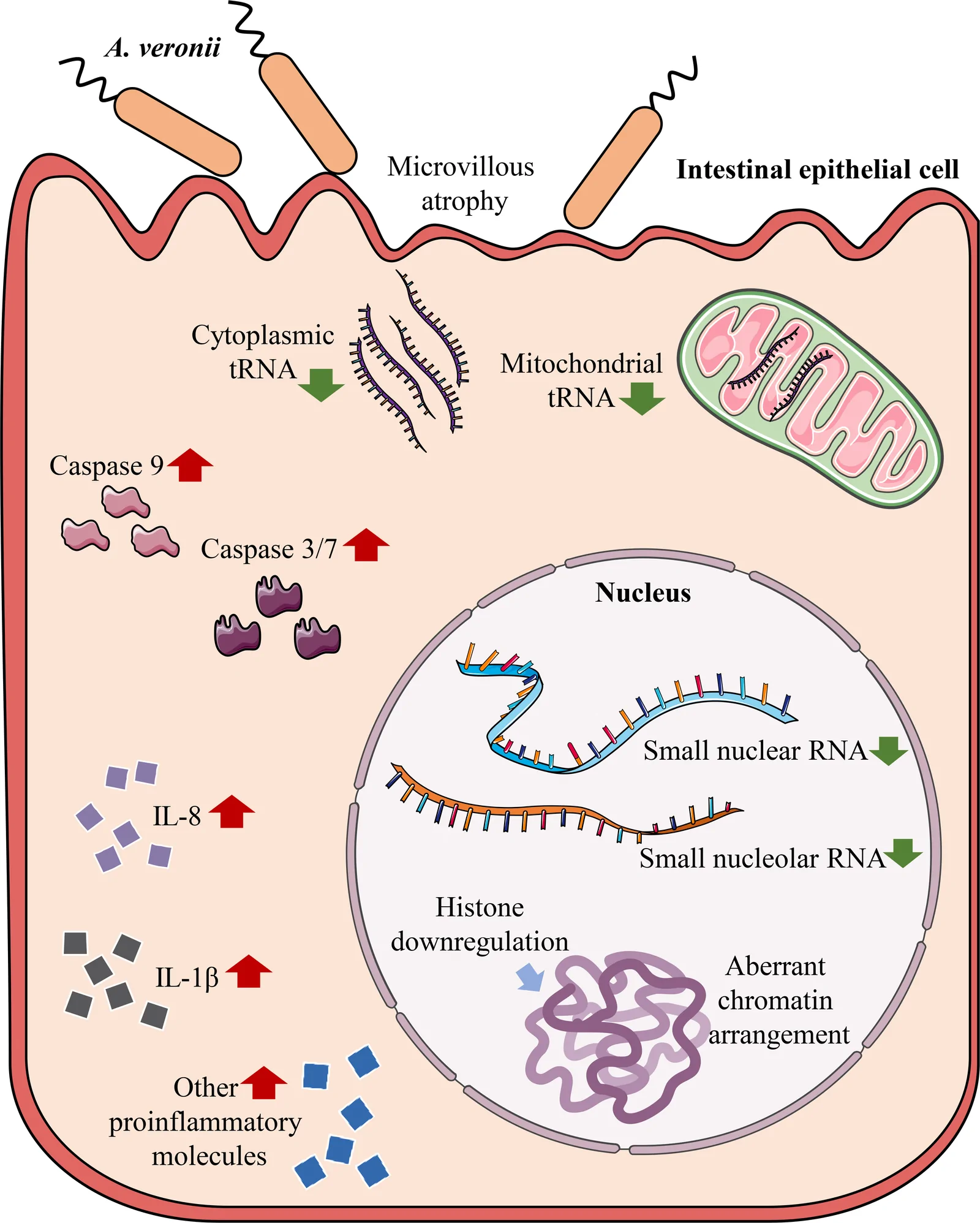
Summary of the pathogenic effects and mechanisms by which *A. veronii* bacteria damage human intestinal epithelial cells. The *A. veronii* AS1 strain induces various detrimental effects in human intestinal epithelial HT-29 cells. These effects include the upregulation of multiple proinflammatory molecules such as IL-1β and IL-8, the downregulation of gene expression of tRNAs, small nuclear RNAs, and small nucleolar RNAs, the downregulation of expression of genes encoding histone proteins, the induction of epithelial cell apoptosis through the intrinsic pathway by increasing caspase 3/7 and caspase 9 activities, the adherence to intestinal epithelial cells, the promotion of microvillous atrophy, and the disruption of chromatin arrangement leading to aberrant patterns.

In summary, we for the first time report an association between the prevalence of *Aeromonas* enteric infection and IBD using bacterial isolation from fecal samples. Furthermore, we show that *A. veronii* damages intestinal epithelial cells through multiple mechanisms (Fig. 7), of which the downregulating cytoplasmic tRNA, small nuclear RNA, and small nucleolar RNA are novel bacterial pathogenic mechanisms.

## Data Availability

Complete genome of *A. veronii* AS1 was submitted to NCBI bacterial genome database under accession number CP114182. The RNA-seq data have been submitted to NCBI Gene Expression Omnibus under accession GSE222117.

## SUPPLEMENTAL MATERIAL

Supplementary Materials spectrum.01088-23-s0001.pdf Supplementary Materials including Supplementary Figure 1, Supplementary Table 1 and Supplementary Table 2.

ASM does not own the copyrights to Supplemental Material that may be linked to, or accessed through, an article. The authors have granted ASM a non-exclusive, world-wide license to publish the Supplemental Material files. Please contact the corresponding author directly for reuse.
