# Supplementary material for: Emerging Aeromonas enteric infections: their association with inflammatory bowel disease and novel pathogenic mechanisms
Source: Microbiol Spectr. 2023 Sep 21;11(5):e01088-23. doi: 10.1128/spectrum.01088-23 (PMC10581128; doi:10.1128/spectrum.01088-23)
Supplement: Supplementary Materials — including Supplementary Figure 1, Supplementary Table 1 and Supplementary Table 2. [file spectrum.01088-23-s0001.pdf]

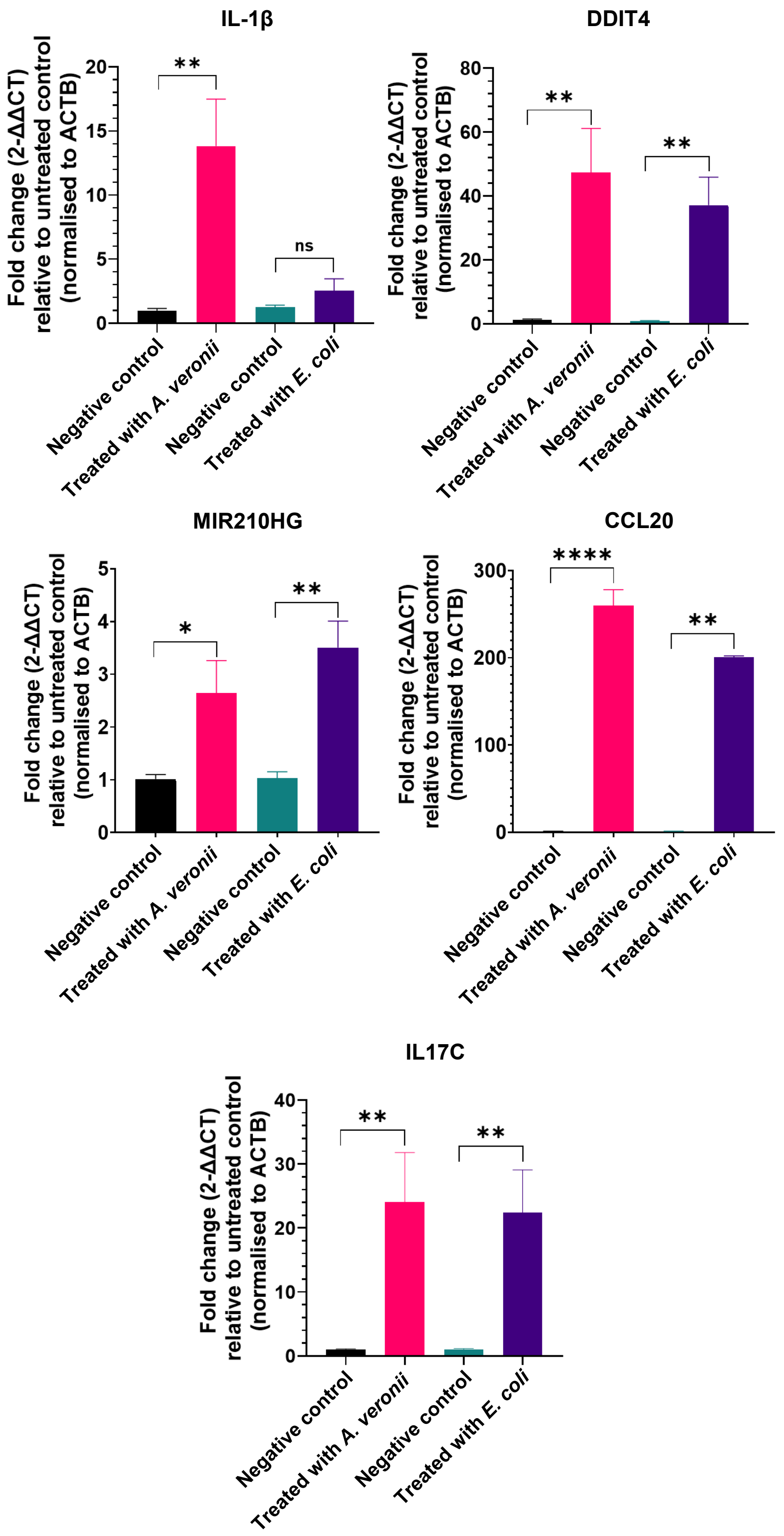

Supplementary Figure 1. Changes of mRNA levels of IL1β, DDIT4, MIR210HG, CCL20, and IL17C in human intestinal epithelial HT-29 cells following *A. veronii* and *E. coli* treatment determined by RT-PCR.

Fold changes were calculated by comparing treated cells to untreated controls which were part of the same quantitative RT-PCR run. Error bars indicate the standard deviation of the mean.

The gene expression levels between groups were compared using unpaired two-tailed *t*-tests. *$p < 0.05$, **$p < 0.01$, ****$p < 0.0001$, and ns > 0.05. Fold changes were normalised to the fold changes of the housekeeping gene ACTB.

Supplementary Table 1. Oligonucleotide primer pairs used in the qRT-PCR experiments for validation

Primers	Sequences (5'→3')	Reference
CCL20	F: CTGGCTGCTTTGATGTCAGT<br>R: CGTGTGAAGCCCACAATAAA	(1)
IL17C	F: CCCTCAGCTACGACCCAGT<br>R: CTTCTGTGGATAGCGGTCCT	(1)
DDIT4	F: CTGGACAGCAGCAACAGTG<br>R: TCACTGAGCAGCTCGAAGTC	(2)
IL-1β	F: GGCCCTAAACAGATGAAGTG<br>R: GTAGTGGTGGTCGGAGATTC	(3)
β-actin	F: ACTCTTCCAGCCTTCCTTC<br>R: GGAGCAATGATCTTGATCTTC	(3)
GAPDH	F: GAGTCAACGGATTTGGTCGT<br>R: TTGATTTTGGAGGGATCTCG	(4)

Supplementary Table 2 Significantly upregulated and downregulated transcripts regulated by *A. veronii* strain AS1 and *E. coli* strain K12.

Gene ID	Gene name	Gene type	Symbol	Log_2 Fold Change	*P*-value	Adjusted *P*-value
	**Significantly upregulated transcripts regulated by *A. veronii* strain AS1**					
3386	intercellular adhesion molecule 4 (Landsteiner-Wiener blood group)	protein-coding	ICAM4	8.2	1.15E-09	1.20E-08
6364	C-C motif chemokine ligand 20	protein-coding	CCL20	7.39	1.07E-109	2.46E-107
53347	ubiquitin associated and SH3 domain containing A	protein-coding	UBASH3A	7.21	1.08E-07	8.82E-07
6703	small proline rich protein 2D	protein-coding	SPRR2D	6.67	1.10E-06	7.62E-06
1440	colony stimulating factor 3	protein-coding	CSF3	5.91	1.79E-05	0.0001018
3624	inhibin subunit beta A	protein-coding	INHBA	5.9	6.01E-09	5.78E-08
27189	interleukin 17C	protein-coding	IL17C	5.39	7.00E-13	1.03E-11
405753	dual oxidase maturation factor 2	protein-coding	DUOXA2	5.12	0.0002257	0.0010408
54541	DNA damage inducible transcript 4	protein-coding	DDIT4	4.98	0	0
3552	interleukin 1 alpha	protein-coding	IL1A	4.78	3.03E-91	5.03E-89
1435	colony stimulating factor 1	protein-coding	CSF1	4.71	2.15E-27	7.84E-26
3383	intercellular adhesion molecule 1	protein-coding	ICAM1	4.43	0	0
3553	interleukin 1 beta	protein-coding	IL1B	4.23	2.12E-32	9.54E-31
6698	small proline rich protein 1A	protein-coding	SPRR1A	4.1	6.36E-51	5.04E-49
1437	colony stimulating factor 2	protein-coding	CSF2	4.04	9.56E-33	4.44E-31
2919	C-X-C motif chemokine ligand 1	protein-coding	CXCL1	4.02	0	0
51129	angiopoietin like 4	protein-coding	ANGPTL4	3.99	3.79E-40	2.29E-38
3604	TNF receptor superfamily member 9	protein-coding	TNFRSF9	3.99	8.62E-12	1.13E-10
7127	TNF alpha induced protein 2	protein-coding	TNFAIP2	3.94	0	0
7130	TNF alpha induced protein 6	protein-coding	TNFAIP6	3.91	3.60E-11	4.47E-10
4322	matrix metallopeptidase 13	protein-coding	MMP13	3.8	1.66E-07	1.32E-06
56892	transcriptional and immune response regulator	protein-coding	TCIM	3.6	0.0009351	0.0037814
6376	C-X3-C motif chemokine ligand 1	protein-coding	CX3CL1	3.47	7.58E-12	1.00E-10
6367	C-C motif chemokine ligand 22	protein-coding	CCL22	3.44	0.0001016	0.0005036
3576	C-X-C motif chemokine ligand 8	protein-coding	CXCL8	3.31	1.63E-269	1.03E-266
27289	Rho family GTPase 1	protein-coding	RND1	3.23	3.10E-167	1.15E-164
2920	C-X-C motif chemokine ligand 2	protein-coding	CXCL2	3.18	7.17E-69	8.55E-67
8676	syntaxin 11	protein-coding	STX11	3.18	0.0002608	0.0011879
8553	basic helix-loop-helix family member e40	protein-coding	BHLHE40	3.13	1.42E-269	9.37E-267
3249	hepsin	protein-coding	HPN	3.1	0.0014489	0.0056075
27076	LY6/PLAUR domain containing 3	protein-coding	LYPD3	3.07	0.0001021	0.0005058
2921	C-X-C motif chemokine ligand 3	protein-coding	CXCL3	2.99	9.98E-105	2.14E-102
57561	arrestin domain containing 3	protein-coding	ARRDC3	2.91	0	0
330	baculoviral IAP repeat containing 3	protein-coding	BIRC3	2.87	0	0
79094	ChaC glutathione specific gamma-glutamylcyclotransferase 1	protein-coding	CHAC1	2.81	1.59E-59	1.54E-57
8482	semaphorin 7A (John Milton Hagen blood group)	protein-coding	SEMA7A_1	2.77	5.07E-15	9.04E-14
133	adrenomedullin	protein-coding	ADM	2.63	0	0
2353	Fos proto-oncogene, AP-1 transcription factor subunit	protein-coding	FOS	2.63	0	0
6406	semenogelin 1	protein-coding	SEMG1	2.53	0.0020064	0.0075083
5155	platelet derived growth factor subunit B	protein-coding	PDGFB	2.52	0	0
93082	neuralized E3 ubiquitin protein ligase 3	protein-coding	NEURL3	2.47	1.06E-11	1.38E-10
5054	serpin family E member 1	protein-coding	SERPINE1	2.47	7.87E-30	3.20E-28
9235	interleukin 32	protein-coding	IL32	2.44	8.93E-17	1.79E-15
9751	syntaphilin	protein-coding	SNPH	2.42	1.94E-08	1.74E-07
123745	phospholipase A2 group IVE	protein-coding	PLA2G4E	2.41	0.0007031	0.0029157
639	PR/SET domain 1	protein-coding	PRDM1	2.39	8.09E-30	3.28E-28
84812	phospholipase C delta 4	protein-coding	PLCD4	2.33	0.0009576	0.0038619
6699	small proline rich protein 1B	protein-coding	SPRR1B	2.32	1.39E-43	8.90E-42
57514	Rho GTPase activating protein 31	protein-coding	ARHGAP31	2.32	1.76E-16	3.47E-15
54206	ERBB receptor feedback inhibitor 1	protein-coding	ERRFI1	2.32	0	0
27113	BCL2 binding component 3	protein-coding	BBC3	2.27	5.99E-49	4.53E-47
5266	peptidase inhibitor 3	protein-coding	PI3	2.25	2.91E-17	6.04E-16
7128	TNF alpha induced protein 3	protein-coding	TNFAIP3	2.23	1.11E-169	4.20E-167
5209	6-phosphofructo-2-kinase/fructose-2,6-biphosphatase 3	protein-coding	PFKFB3	2.19	0	0
4791	nuclear factor kappa B subunit 2	protein-coding	NFKB2	2.16	0	0
1.2E+08	uncharacterized LOC124900374	protein-coding	LOC124900374	2.07	1.09E-11	1.41E-10
9518	growth differentiation factor 15	protein-coding	GDF15	2.06	2.26E-237	1.18E-234
624	bradykinin receptor B2	protein-coding	BDKRB2	2.02	1.77E-05	0.0001005
112399	egl-9 family hypoxia inducible factor 3	protein-coding	EGLN3	2.02	0	0
192134	UDP-GlcNAc:betaGal beta-1,3-N-acetylglucosaminyltransferase 6	protein-coding	B3GNT6	1.98	0.0001134	0.0005549
602	BCL3 transcription coactivator	protein-coding	BCL3	1.94	1.49E-290	1.06E-287
3725	Jun proto-oncogene, AP-1 transcription factor subunit	protein-coding	JUN	1.89	0	0
1.1E+08	uncharacterized LOC105376714	protein-coding	LOC105376714	1.86	2.68E-16	5.22E-15
92162	transmembrane protein 88	protein-coding	TMEM88	1.86	3.48E-07	2.62E-06
1026	cyclin dependent kinase inhibitor 1A	protein-coding	CDKN1A	1.85	1.19E-256	7.25E-254
4586	mucin 5AC, oligomeric mucus/gel-forming	protein-coding	MUC5AC_1	1.84	0.0003329	0.001481
10633	RAS like family 10 member A	protein-coding	RASL10A	1.83	1.37E-06	9.40E-06
5743	prostaglandin-endoperoxide synthase 2	protein-coding	PTGS2	1.83	1.46E-276	9.99E-274
5328	plasminogen activator, urokinase	protein-coding	PLAU	1.78	2.23E-27	8.10E-26
4792	NFKB inhibitor alpha	protein-coding	NFKBIA	1.73	4.63E-304	3.58E-301
25841	ankyrin repeat and BTB domain containing 2	protein-coding	ABTB2	1.73	0	0
140453	mucin 17, cell surface associated	protein-coding	MUC17	1.71	1.03E-32	4.76E-31
6926	T-box transcription factor 3	protein-coding	TBX3	1.71	0	0
8876	vanin 1	protein-coding	VNN1	1.7	9.13E-06	5.48E-05

12	serpin family A member 3	protein-coding	SERPINA3	1.69	2.35E-21	6.17E-20
23135	lysine demethylase 6B	protein-coding	KDM6B	1.68	4.31E-68	5.00E-66
9308	CD83 molecule	protein-coding	CD83	1.65	2.26E-11	2.86E-10
1316	KLF transcription factor 6	protein-coding	KLF6	1.64	4.43E-48	3.28E-46
7584	zinc finger protein 35	protein-coding	ZNF35	1.64	0.000106	0.0005222
6274	S100 calcium binding protein A3	protein-coding	S100A3	1.64	5.78E-23	1.66E-21
5293	phosphatidylinositol-4,5-bisphosphate 3-kinase catalytic subunit delta	protein-coding	PIK3CD	1.63	3.16E-11	3.95E-10
7422	vascular endothelial growth factor A	protein-coding	VEGFA	1.62	0	0
389384	BCL2 interacting protein 5	protein-coding	BNIP5	1.62	6.89E-10	7.38E-09
1999	E74 like ETS transcription factor 3	protein-coding	ELF3	1.61	4.09E-292	3.03E-289
3038	hyaluronan synthase 3	protein-coding	HAS3	1.6	1.11E-153	3.63E-151
1051	CCAAT enhancer binding protein beta	protein-coding	CEBPB	1.58	1.39E-225	6.84E-223
1.2E+08	translation initiation factor IF-2-like	protein-coding	LOC124904106	1.55	0.0024485	0.008983
9099	ubiquitin specific peptidase 2	protein-coding	USP2	1.54	2.10E-19	4.97E-18
2354	FosB proto-oncogene, AP-1 transcription factor subunit	protein-coding	FOSB	1.53	1.49E-46	1.04E-44
651746	ankyrin repeat domain 33B	protein-coding	ANKRD33B	1.53	2.68E-07	2.05E-06
22822	pleckstrin homology like domain family A member 1	protein-coding	PHLDA1	1.52	1.89E-240	1.02E-237
10957	proline rich nuclear receptor coactivator 1	protein-coding	PNRC1	1.52	1.03E-103	2.15E-101
163175	leucine rich repeat LGI family member 4	protein-coding	LGI4	1.51	0.0012214	0.0048057
10397	N-myc downstream regulated 1	protein-coding	NDRG1	1.51	1.61E-250	9.51E-248
694	BTG anti-proliferation factor 1	protein-coding	BTG1	1.5	3.13E-213	1.39E-210
162394	schlafen family member 5	protein-coding	SLFN5	1.5	1.37E-14	2.37E-13
131583	family with sequence similarity 43 member A	protein-coding	FAM43A	1.47	3.85E-26	1.29E-24
3460	interferon gamma receptor 2	protein-coding	IFNGR2	1.47	2.04E-10	2.33E-09
6707	small proline rich protein 3	protein-coding	SPRR3	1.47	8.61E-07	6.09E-06
2669	GTP binding protein overexpressed in skeletal muscle	protein-coding	GEM	1.47	1.30E-53	1.10E-51
23150	FERM domain containing 4B	protein-coding	FRMD4B	1.46	1.95E-58	1.85E-56
79924	adrenomedullin 2	protein-coding	ADM2	1.42	2.50E-22	6.93E-21
3656	interleukin 1 receptor associated kinase 2	protein-coding	IRAK2	1.42	3.18E-149	1.01E-146
90427	Bcl2 modifying factor	protein-coding	BMF	1.42	1.70E-67	1.96E-65
222611	adhesion G protein-coupled receptor F2	protein-coding	ADGRF2	1.42	2.07E-16	4.05E-15
6700	small proline rich protein 2A	protein-coding	SPRR2A	1.4	0.0075234	0.0242137
1942	ephrin A1	protein-coding	EFNA1	1.4	7.96E-122	2.05E-119
6385	syndecan 4	protein-coding	SDC4	1.4	0	0
8013	nuclear receptor subfamily 4 group A member 3	protein-coding	NR4A3	1.39	0.0007643	0.0031469
23780	apolipoprotein L2	protein-coding	APOL2	1.38	1.18E-127	3.24E-125
1847	dual specificity phosphatase 5	protein-coding	DUSP5	1.37	2.53E-101	5.00E-99
9734	histone deacetylase 9	protein-coding	HDAC9	1.37	2.84E-10	3.20E-09
11074	tripartite motif containing 31	protein-coding	TRIM31	1.37	5.03E-06	3.15E-05
8743	TNF superfamily member 10	protein-coding	TNFSF10	1.36	3.53E-34	1.72E-32
313	acyloxyacyl hydrolase	protein-coding	AOAH	1.35	0.0053102	0.0178547
80117	ADP ribosylation factor like GTPase 14	protein-coding	ARL14	1.35	3.07E-182	1.21E-179
80149	zinc finger CCCH-type containing 12A	protein-coding	ZC3H12A	1.34	4.30E-165	1.56E-162
1839	heparin binding EGF like growth factor	protein-coding	HBEGF	1.34	3.27E-47	2.35E-45
114907	F-box protein 32	protein-coding	FBXO32	1.34	5.19E-77	7.20E-75
163259	DENN domain containing 2C	protein-coding	DENND2C	1.33	1.35E-14	2.33E-13
81788	NUAK family kinase 2	protein-coding	NUAK2	1.33	6.10E-12	8.14E-11
901	cyclin G2	protein-coding	CCNG2	1.32	3.25E-146	9.94E-144
23138	NEDD4 binding protein 3	protein-coding	N4BP3	1.31	3.33E-42	2.09E-40
9314	KLF transcription factor 4	protein-coding	KLF4	1.31	2.74E-221	1.31E-218
80271	inositol-trisphosphate 3-kinase C	protein-coding	ITPKC	1.3	9.09E-127	2.41E-124
1907	endothelin 2	protein-coding	EDN2	1.28	0.0077783	0.0249121
5971	RELB proto-oncogene, NF-kB subunit	protein-coding	RELB	1.27	3.29E-112	8.11E-110
10509	semaphorin 4B	protein-coding	SEMA4B	1.26	6.46E-246	3.70E-243
5329	plasminogen activator, urokinase receptor	protein-coding	PLAUR	1.24	3.36E-183	1.35E-180
10435	CDC42 effector protein 2	protein-coding	CDC42EP2	1.24	1.30E-201	5.51E-199
25833	POU class 2 homeobox 3	protein-coding	POU2F3	1.23	0.0032265	0.0114721
84830	androgen dependent TFPI regulating protein	protein-coding	ADTRP	1.23	0.0015547	0.0059792
467	activating transcription factor 3	protein-coding	ATF3	1.22	1.92E-27	7.03E-26
4665	NGFI-A binding protein 2	protein-coding	NAB2	1.21	1.25E-101	2.52E-99
90874	zinc finger protein 697	protein-coding	ZNF697	1.2	1.23E-34	6.13E-33
84002	UDP-GlcNAc:betaGal beta-1,3-N-acetylglucosaminyltransferase 5	protein-coding	B3GNT5	1.2	8.20E-212	3.55E-209
3097	HIVEP zinc finger 2	protein-coding	HIVEP2	1.2	4.76E-125	1.24E-122
64699	transmembrane serine protease 3	protein-coding	TMPRSS3	1.2	0.0044019	0.0151215
6892	TAP binding protein	protein-coding	TAPBP_3	1.19	0.0004214	0.0018349
6591	snail family transcriptional repressor 2	protein-coding	SNAI2	1.19	0.0020812	0.007762
113791	phosphoinositide-3-kinase interacting protein 1	protein-coding	PIK3IP1	1.18	6.45E-05	0.0003319
8542	apolipoprotein L1	protein-coding	APOL1	1.18	8.22E-36	4.23E-34
1E+08	BLACAT1 overlapping LEMD1 locus	protein-coding	BLACAT1	1.18	2.23E-29	8.97E-28
3549	Indian hedgehog signaling molecule	protein-coding	IHH	1.17	8.45E-65	9.09E-63
55818	lysine demethylase 3A	protein-coding	KDM3A	1.17	3.50E-128	9.70E-126
23645	protein phosphatase 1 regulatory subunit 15A	protein-coding	PPP1R15A	1.16	6.93E-108	1.56E-105
353322	ankyrin repeat domain 37	protein-coding	ANKRD37	1.16	2.07E-23	6.01E-22
6776	signal transducer and activator of transcription 5A	protein-coding	STAT5A	1.15	6.21E-23	1.78E-21
54498	spermine oxidase	protein-coding	SMOX	1.14	6.84E-242	3.80E-239
374	amphiregulin	protein-coding	AREG	1.13	5.80E-180	2.24E-177
642273	family with sequence similarity 110 member C	protein-coding	FAM110C	1.12	6.86E-82	9.98E-80
2529	fucosyltransferase 7	protein-coding	FUT7	1.12	0.0020864	0.0077798
677	ZFP36 ring finger protein like 1	protein-coding	ZFP36L1	1.11	1.91E-143	5.76E-141
3726	JunB proto-oncogene, AP-1 transcription factor subunit	protein-coding	JUNB	1.11	6.43E-131	1.81E-128
254439	chromosome 11 open reading frame 86	protein-coding	C11orf86	1.1	8.89E-09	8.32E-08
652968	cytosolic arginine sensor for mTORC1 subunit 1	protein-coding	CASTOR1	1.08	0.0011844	0.0046757

1647	growth arrest and DNA damage inducible alpha	protein-coding	GADD45A	1.07	7.07E-33	3.31E-31
285513	GPRIN family member 3	protein-coding	GPRIN3	1.07	3.48E-11	4.33E-10
91828	exocyst complex component 3 like 4	protein-coding	EXOC3L4	1.06	0.0026934	0.0097743
6498	SKI like proto-oncogene	protein-coding	SKIL	1.06	2.21E-183	9.13E-181
2069	epiregulin	protein-coding	EREG	1.05	1.98E-233	1.00E-230
5966	REL proto-oncogene, NF-kB subunit	protein-coding	REL	1.05	3.08E-89	4.97E-87
55344	phosphatidylinositol specific phospholipase C X domain containing 1	protein-coding	PLCXD1_1	1.05	0.0015295	0.0058915
4739	neural precursor cell expressed, developmentally down-regulated 9	protein-coding	NEDD9	1.05	2.17E-110	5.20E-108
4794	NFKB inhibitor epsilon	protein-coding	NFKBIE	1.05	6.56E-48	4.81E-46
1848	dual specificity phosphatase 6	protein-coding	DUSP6	1.04	1.55E-133	4.45E-131
390637	GDP-D-glucose phosphorylase 1	protein-coding	GDPGP1	1.04	7.16E-59	6.87E-57
64651	cysteine and serine rich nuclear protein 1	protein-coding	CSRNP1	1.04	2.94E-49	2.28E-47
163732	Cbp/p300 interacting transactivator with Glu/Asp rich carboxy-terminal domain 4	protein-coding	CITED4	1.03	7.11E-27	2.49E-25
440307	tubulin tyrosine ligase like 13	protein-coding	TTLL13	1.03	2.46E-05	0.0001362
8795	TNF receptor superfamily member 10b	protein-coding	TNFRSF10B	1.03	4.86E-220	2.27E-217
1960	early growth response 3	protein-coding	EGR3	1.03	0.0026029	0.0094787
5272	serpin family B member 9	protein-coding	SERPINB9	1.02	3.72E-30	1.53E-28
4616	growth arrest and DNA damage inducible beta	protein-coding	GADD45B	1.02	4.88E-22	1.33E-20
4485	macrophage stimulating 1	protein-coding	MST1	1.02	0.0004389	0.0019043
50506	dual oxidase 2	protein-coding	DUOX2	1.01	0.0013626	0.0053108
79025	fibronectin type III domain containing 11	protein-coding	FNDC11	1	6.41E-05	0.0003303
1.1E+08	MIR3142 host gene	ncRNA	MIR3142HG	5.29	0.0001212	0.0005904
1E+08	eosinophil granule ontogeny transcript	ncRNA	EGOT	5.25	0.0001303	0.0006311
1E+08	uncharacterized LOC101928554	ncRNA	LOC101928554	4.61	6.96E-06	4.26E-05
1.1E+08	uncharacterized LOC105375914	ncRNA	LOC105375914	3.72	0.0007353	0.0030382
1.2E+08	uncharacterized LOC124902449	ncRNA	LOC124902449	3.29	6.50E-10	6.99E-09
1.1E+08	uncharacterized LOC105371159	ncRNA	LOC105371159	3.26	1.43E-23	4.20E-22
1.1E+08	uncharacterized LOC107984192	ncRNA	LOC107984192	2.49	0.0010319	0.004131
1.1E+08	uncharacterized LOC107984862	ncRNA	LOC107984862	2.32	3.11E-05	0.0001698
1E+08	MIR210 host gene	ncRNA	MIR210HG_1	2.1	5.97E-19	1.37E-17
1E+08	lung cancer associated transcript 1	ncRNA	LUCAT1	2.09	8.85E-95	1.54E-92
1.1E+08	uncharacterized LOC107985284	ncRNA	LOC107985284	2.09	3.78E-14	6.24E-13
1E+08	HIF1A antisense RNA 2	ncRNA	HIF1A-AS2	2	1.02E-09	1.08E-08
1.2E+08	uncharacterized LOC124902540	ncRNA	LOC124902540	1.94	0.0016776	0.0064021
1.2E+08	uncharacterized LOC124902793	ncRNA	LOC124902793	1.88	0.0004873	0.0020945
1.2E+08	uncharacterized LOC124903044	ncRNA	LOC124903044	1.65	0.0003543	0.0015671
400798	chromosome 1 putative open reading frame 220	ncRNA	C1orf220	1.65	5.79E-10	6.26E-09
1.1E+08	uncharacterized LOC105376361	ncRNA	LOC105376361	1.57	0.0044486	0.015261
728228	long intergenic non-protein coding RNA 1433	ncRNA	LINC01433	1.56	1.14E-08	1.05E-07
1.2E+08	uncharacterized LOC124902691	ncRNA	LOC124902691	1.55	2.06E-61	2.06E-59
1.1E+08	EGLN3 antisense RNA 1	ncRNA	EGLN3-AS1	1.36	0.0010891	0.0043321
1.1E+08	uncharacterized LOC105369344	ncRNA	LOC105369344	1.32	4.09E-30	1.68E-28
1E+08	uncharacterized LOC102724965	ncRNA	LOC102724965	1.26	0.0005152	0.002195
399715	long intergenic non-protein coding RNA 2649	ncRNA	LINC02649	1.25	0.003303	0.0117112
253018	HLA complex group 27	ncRNA	HCG27_4	1.18	0.0040468	0.01404
1.1E+08	HIVEP2 divergent transcript	ncRNA	HIVEP2-DT	1.17	0.0010491	0.0041889
1.1E+08	GAPDH divergent transcript	ncRNA	GAPDH-DT	1.16	7.56E-05	0.0003832
1.2E+08	uncharacterized LOC124902276	ncRNA	LOC124902276	1.13	3.88E-06	2.48E-05
1.1E+08	uncharacterized LOC105374852	ncRNA	LOC105374852	1.12	1.55E-09	1.60E-08
1.2E+08	ERRFI1 divergent transcript	ncRNA	ERRFI1-DT	1.11	0.0007176	0.0029708
1E+08	SPACA6 antisense RNA 1	ncRNA	SPACA6-AS1	1.1	2.29E-06	1.51E-05
1.1E+08	TPM1 antisense RNA	ncRNA	TPM1-AS	1.07	0.0005557	0.0023507
1E+08	uncharacterized LOC102724351	ncRNA	LOC102724351	1.05	0.0005137	0.0021908
1.1E+08	uncharacterized LOC105369299	ncRNA	LOC105369299	1.04	0.0010241	0.0041031
1E+08	microRNA 3648-2	ncRNA	MIR3648-2	1.04	0.0004252	0.0018499
1.1E+08	ARHGEF2 antisense RNA 2	ncRNA	ARHGEF2-AS2	1.03	0.0005939	0.0025004
1.1E+08	uncharacterized LOC105370672	ncRNA	LOC105370672	1.02	1.82E-08	1.65E-07
1E+08	cell division cycle 27 pseudogene 10	pseudogene	CDC27P10	3.91	0.0005418	0.002297
340198	interferon induced transmembrane protein 4 pseudogene	pseudogene	IFITM4P_1	2.21	0.000833	0.0034044
157489	SDA1 domain containing 1 pseudogene 1	pseudogene	SDAD1P1	1.24	0.0014045	0.0054571
1E+08	clustered mitochondria homolog pseudogene 3	pseudogene	CLUHP3	1.21	1.69E-30	7.06E-29
**Significantly downregulated transcripts regulated by *A. veronii* strain AS1**						
4696	NADH:ubiquinone oxidoreductase subunit A3	protein-coding	NDUFA3	-2.9	0.0001874	0.0008806
389058	Sp5 transcription factor	protein-coding	SP5	-2.64	8.49E-21	2.17E-19
10628	thioredoxin interacting protein	protein-coding	TXNIP	-2.52	0	0
1.1E+08	uncharacterized LOC105369669	protein-coding	LOC105369669	-2.43	4.10E-11	5.04E-10
4541	NADH dehydrogenase subunit 6	protein-coding	ND6	-2.29	0	0
646862	ring finger protein 225	protein-coding	RNF225	-2.05	2.30E-06	1.52E-05
1.2E+08	uncharacterized LOC124903317	protein-coding	LOC124903317	-1.92	0.0043722	0.015031
51421	angiomotin like 2	protein-coding	AMOTL2	-1.77	0	0
63939	family with sequence similarity 217 member B	protein-coding	FAM217B	-1.75	1.86E-101	3.71E-99
8313	axin 2	protein-coding	AXIN2	-1.66	6.71E-214	3.06E-211
57574	membrane associated ring-CH-type finger 4	protein-coding	MARCHF4	-1.58	1.03E-06	7.22E-06
1.2E+08	uncharacterized LOC124902530	protein-coding	LOC124902530	-1.52	0.0025786	0.0094057
3310	heat shock protein family A (Hsp70) member 6	protein-coding	HSPA6	-1.49	1.99E-16	3.89E-15
25937	WW domain containing transcription regulator 1	protein-coding	WWTR1	-1.48	1.68E-05	9.59E-05
8851	cyclin dependent kinase 5 regulatory subunit 1	protein-coding	CDK5R1	-1.46	2.01E-41	1.24E-39
91947	arrestin domain containing 4	protein-coding	ARRDC4	-1.41	5.71E-115	1.43E-112
3670	ISL LIM homeobox 1	protein-coding	ISL1	-1.4	6.91E-16	1.31E-14
8356	H3 clustered histone 12	protein-coding	H3C12	-1.39	3.13E-154	1.05E-151
116039	odd-skipped related transciption factor 2	protein-coding	OSR2	-1.38	1.75E-11	2.23E-10
8462	KLF transcription factor 11	protein-coding	KLF11	-1.35	2.38E-67	2.73E-65

1983	eukaryotic translation initiation factor 5	protein-coding	EIF5	-1.34	0	0
84189	SLIT and NTRK like family member 6	protein-coding	SLITRK6	-1.32	9.32E-22	2.50E-20
51306	family with sequence similarity 13 member B	protein-coding	FAM13B	-1.27	5.27E-121	1.34E-118
8350	H3 clustered histone 1	protein-coding	H3C1	-1.26	2.27E-23	6.60E-22
79852	epoxide hydrolase 3	protein-coding	EPHX3	-1.23	0.0074121	0.0238989
55727	BTB domain containing 7	protein-coding	BTBD7	-1.22	0.0026649	0.0096846
89890	kelch repeat and BTB domain containing 6	protein-coding	KBTBD6	-1.22	2.72E-57	2.52E-55
8970	H2B clustered histone 11	protein-coding	H2BC11	-1.22	2.37E-36	1.24E-34
84206	mex-3 RNA binding family member B	protein-coding	MEX3B	-1.2	7.82E-05	0.0003954
8335	H2A clustered histone 4	protein-coding	H2AC4	-1.2	1.91E-76	2.59E-74
121504	H4 histone 16	protein-coding	H4-16	-1.18	6.75E-101	1.30E-98
653604	H3 clustered histone 13	protein-coding	H3C13	-1.14	2.46E-109	5.60E-107
8366	H4 clustered histone 2	protein-coding	H4C2	-1.14	6.52E-26	2.15E-24
1.2E+08	uncharacterized LOC124903219	protein-coding	LOC124903219	-1.13	5.48E-11	6.64E-10
8352	H3 clustered histone 3	protein-coding	H3C3	-1.13	1.69E-82	2.49E-80
79960	jade family PHD finger 1	protein-coding	JADE1	-1.13	5.01E-50	3.91E-48
91461	protein kinase domain containing, cytoplasmic	protein-coding	PKDCC	-1.13	2.22E-163	7.87E-161
56169	gasdermin C	protein-coding	GSDMC	-1.13	0.001789	0.0067705
8330	H2A clustered histone 15	protein-coding	H2AC15	-1.12	5.34E-46	3.72E-44
1844	dual specificity phosphatase 2	protein-coding	DUSP2	-1.09	2.37E-37	1.29E-35
84733	chromobox 2	protein-coding	CBX2	-1.09	2.43E-20	6.08E-19
144165	prickle planar cell polarity protein 1	protein-coding	PRICKLE1	-1.07	0.0015424	0.0059345
118738	zinc finger protein 488	protein-coding	ZNF488	-1.06	6.68E-55	5.78E-53
7023	transcription factor AP-4	protein-coding	TFAP4	-1.06	1.25E-60	1.22E-58
201164	phospholipase D family member 6	protein-coding	PLD6	-1.05	6.50E-14	1.06E-12
55113	XK related 8	protein-coding	XKR8	-1.05	4.05E-14	6.68E-13
8332	H2A clustered histone 16	protein-coding	H2AC16	-1.04	3.55E-90	5.79E-88
1.2E+08	uncharacterized LOC124906092	protein-coding	LOC124906092	-1.03	1.45E-10	1.68E-09
91893	ferredoxin-fold anticodon binding domain containing 1	protein-coding	FDXACB1	-1.03	2.41E-17	5.02E-16
401027	chromosome 2 open reading frame 66	protein-coding	C2orf66	-1.02	0.0039722	0.0138038
7042	transforming growth factor beta 2	protein-coding	TGFB2	-1.01	7.69E-10	8.19E-09
90075	zinc finger protein 30	protein-coding	ZNF30	-1	8.85E-14	1.42E-12
1.1E+08	uncharacterized LOC105378198	ncRNA	LOC105378198	-4.35	0.0001075	0.0005287
56662	vault RNA 1-3	ncRNA	VTRNA1-3	-4.01	2.69E-12	3.74E-11
1.1E+08	uncharacterized LOC105372436	ncRNA	LOC105372436	-3.68	0.0007239	0.0029957
1.1E+08	uncharacterized LOC107986401	ncRNA	LOC107986401	-3.27	2.00E-07	1.57E-06
1.1E+08	long intergenic non-protein coding RNA 2739	ncRNA	LINC02739	-2.89	1.47E-05	8.47E-05
407014	microRNA 25	ncRNA	MIR25	-2.77	1.52E-05	8.78E-05
407975	miR-17-92a-1 cluster host gene	ncRNA	MIR17HG	-2.64	4.67E-148	1.45E-145
574036	SERTAD4 antisense RNA 1	ncRNA	SERTAD4-AS1	-2.56	0.0039163	0.0136247
1E+08	microRNA 5087	ncRNA	MIR5087	-2.39	0.0001496	0.0007142
406886	microRNA let-7d	ncRNA	MIRLET7D	-2.23	0.003484	0.0122769
693233	microRNA 648	ncRNA	MIR648	-2.05	0.001982	0.0074235
1E+08	microRNA 5047	ncRNA	MIR5047	-1.75	3.05E-25	9.73E-24
1.2E+08	uncharacterized LOC124904207	ncRNA	LOC124904207	-1.75	2.27E-07	1.77E-06
1.2E+08	uncharacterized LOC124903222	ncRNA	LOC124903222	-1.72	0.0005237	0.0022289
1.2E+08	uncharacterized LOC124903536	ncRNA	LOC124903536	-1.72	2.99E-08	2.61E-07
1.2E+08	uncharacterized LOC124901940	ncRNA	LOC124901940	-1.7	0.0046645	0.0159094
1E+08	long intergenic non-protein coding RNA 2728	ncRNA	LINC02728	-1.58	0.0021248	0.0079082
1.2E+08	uncharacterized LOC124903201	ncRNA	LOC124903201	-1.55	0.0001951	0.0009117
1.2E+08	uncharacterized LOC124902194	ncRNA	LOC124902194	-1.53	1.94E-07	1.52E-06
1.2E+08	uncharacterized LOC124904895	ncRNA	LOC124904895	-1.52	8.45E-11	1.00E-09
1.1E+08	pre-mRNA-splicing factor cwc22-like	ncRNA	LOC107985388	-1.52	5.56E-14	9.10E-13
1.1E+08	uncharacterized LOC105377310	ncRNA	LOC105377310	-1.52	1.06E-06	7.41E-06
1.2E+08	uncharacterized LOC124905009	ncRNA	LOC124905009	-1.45	0.0044377	0.0152326
1.2E+08	uncharacterized LOC124900477	ncRNA	LOC124900477	-1.41	1.44E-09	1.49E-08
1.2E+08	uncharacterized LOC124902655	ncRNA	LOC124902655	-1.39	5.06E-05	0.0002659
574406	ADAMTSL4 antisense RNA 1	ncRNA	ADAMTSL4-AS1	-1.39	6.94E-05	0.0003551
1.1E+08	MRAP antisense RNA 1	ncRNA	MRAP-AS1	-1.33	1.73E-07	1.37E-06
1.1E+08	uncharacterized LOC105379280	ncRNA	LOC105379280	-1.31	6.28E-18	1.35E-16
1.2E+08	uncharacterized LOC124902961	ncRNA	LOC124902961	-1.3	7.98E-07	5.68E-06
1.2E+08	uncharacterized LOC124906309	ncRNA	LOC124906309	-1.29	6.64E-06	4.07E-05
731075	uncharacterized LOC731075	ncRNA	LOC731075	-1.29	6.24E-07	4.52E-06
1.2E+08	uncharacterized LOC124903326	ncRNA	LOC124903326	-1.25	0.0008339	0.0034063
1E+08	LIX1 and RIOK2 antisense RNA 1	ncRNA	LIX1-AS1	-1.25	0.0035049	0.0123359
1.1E+08	uncharacterized LOC105369477	ncRNA	LOC105369477	-1.24	6.31E-08	5.29E-07
1.2E+08	uncharacterized LOC124901767	ncRNA	LOC124901767	-1.23	0.0022383	0.0082801
1.1E+08	uncharacterized LOC107985684	ncRNA	LOC107985684	-1.22	4.07E-07	3.04E-06
1.1E+08	uncharacterized LOC105374711	ncRNA	LOC105374711	-1.22	3.95E-10	4.37E-09
1.2E+08	uncharacterized LOC124902979	ncRNA	LOC124902979	-1.22	1.17E-07	9.48E-07
1.2E+08	uncharacterized LOC124903631	ncRNA	LOC124903631	-1.17	2.70E-15	4.89E-14
1.2E+08	uncharacterized LOC124900987	ncRNA	LOC124900987	-1.12	2.25E-05	0.0001257
1.1E+08	uncharacterized LOC105371622	ncRNA	LOC105371622	-1.11	0.0004939	0.0021204
1E+08	uncharacterized LOC100996756	ncRNA	LOC100996756	-1.11	3.53E-05	0.0001903
1.2E+08	uncharacterized LOC124905127	ncRNA	LOC124905127	-1.11	0.0001046	0.0005166
1E+08	long intergenic non-protein coding RNA 1424	ncRNA	LINC01424	-1.1	0.004284	0.0147649
1.2E+08	uncharacterized LOC124904901	ncRNA	LOC124904901	-1.09	4.34E-05	0.0002303
1E+08	uncharacterized LOC100132356	ncRNA	LOC100132356	-1.07	3.76E-08	3.25E-07
1.1E+08	miRlet-7a-1/let-7f-1/let-7d cluster host gene	ncRNA	MIRLET7A1HG	-1.07	9.13E-06	5.48E-05
1.2E+08	uncharacterized LOC124904855	ncRNA	LOC124904855	-1.07	8.33E-05	0.0004186
1.2E+08	uncharacterized LOC124901304	ncRNA	LOC124901304	-1.06	1.54E-05	8.87E-05
1.2E+08	uncharacterized LOC124901769	ncRNA	LOC124901769	-1.02	0.0046004	0.0157211

401261	uncharacterized LOC401261	ncRNA	LOC401261	-1.02	1.21E-07	9.79E-07
339263	long intergenic non-protein coding RNA 2693	ncRNA	LINC02693	-1.01	1.00E-12	1.44E-11
1.2E+08	uncharacterized LOC124905199	ncRNA	LOC124905199	-1	5.02E-05	0.0002642
1E+08	RNA, variant U1 small nuclear 6	snRNA	RNVU1-6	-3.26	1.47E-06	1.01E-05
26828	RNA, U5F small nuclear 1	snRNA	RNU5F-1	-2.91	4.23E-07	3.15E-06
1.2E+08	U1 spliceosomal RNA	snRNA	LOC124904627	-2.59	3.44E-07	2.59E-06
1E+08	RNA, variant U1 small nuclear 8	snRNA	RNVU1-8	-2.29	1.38E-05	8.00E-05
1.2E+08	U1 spliceosomal RNA	snRNA	LOC124904621	-2.15	1.16E-10	1.36E-09
1E+08	RNA, variant U1 small nuclear 15	snRNA	RNVU1-15	-2.08	3.28E-05	0.0001785
1.2E+08	U1 spliceosomal RNA	snRNA	LOC124904613	-1.41	2.18E-06	1.45E-05
26863	RNA, variant U1 small nuclear 18	snRNA	RNVU1-18	-1.33	0.0029243	0.0105114
1E+08	RNA, variant U1 small nuclear 14	snRNA	RNVU1-14	-1.3	3.16E-07	2.39E-06
1E+08	RNA, U6 small nuclear 2	snRNA	RNU6-2	-1.28	1.35E-07	1.08E-06
1.2E+08	U2 spliceosomal RNA	snRNA	LOC124904138	-1.26	3.29E-11	4.10E-10
26831	RNA, U5A small nuclear 1	snRNA	RNU5A-1	-1.12	5.04E-07	3.71E-06
654321	small nucleolar RNA, H/ACA box 75	snoRNA	SNORA75	-3.95	4.16E-15	7.45E-14
1.2E+08	small nucleolar RNA U13	snoRNA	LOC124903799	-3.09	0.0012695	0.0049819
1.2E+08	small nucleolar RNA SNORA81	snoRNA	LOC124900469	-2.9	0.0001785	0.0008419
677831	small nucleolar RNA, H/ACA box 51	snoRNA	SNORA51	-2.87	4.59E-13	6.92E-12
692198	small nucleolar RNA, C/D box 78	snoRNA	SNORD78	-2.39	1.40E-07	1.12E-06
85391	small nucleolar RNA, C/D box 14E	snoRNA	SNORD14E	-2.28	2.61E-25	8.32E-24
1.2E+08	small nucleolar RNA SNORD22	snoRNA	LOC124900306	-2.05	8.76E-15	1.54E-13
26765	small nucleolar RNA, C/D box 12C	snoRNA	SNORD12C	-1.97	1.06E-12	1.53E-11
677812	small nucleolar RNA, H/ACA box 29	snoRNA	SNORA29	-1.94	0.0009246	0.0037452
85390	small nucleolar RNA, C/D box 14D	snoRNA	SNORD14D	-1.82	2.17E-07	1.69E-06
26806	small nucleolar RNA, C/D box 44	snoRNA	SNORD44	-1.78	5.36E-12	7.19E-11
692058	small nucleolar RNA, C/D box 11	snoRNA	SNORD11	-1.77	4.77E-06	3.00E-05
9300	small nucleolar RNA, C/D box 28	snoRNA	SNORD28	-1.77	1.17E-13	1.85E-12
1.2E+08	small nucleolar RNA U13	snoRNA	LOC124905074	-1.76	0.0005148	0.0021936
677792	small nucleolar RNA, H/ACA box 1	snoRNA	SNORA1	-1.73	2.97E-19	6.96E-18
677806	small nucleolar RNA, H/ACA box 20	snoRNA	SNORA20	-1.72	0.0012998	0.0050871
1.2E+08	small nucleolar RNA U13	snoRNA	LOC124902586	-1.7	0.0037161	0.0129916
677850	small nucleolar RNA, C/D box 1C	snoRNA	SNORD1C	-1.69	1.77E-06	1.19E-05
692075	small nucleolar RNA, C/D box 6	snoRNA	SNORD6	-1.64	9.75E-09	9.10E-08
9298	small nucleolar RNA, C/D box 31	snoRNA	SNORD31	-1.63	7.19E-20	1.76E-18
26793	small nucleolar RNA, C/D box 56	snoRNA	SNORD56	-1.57	0.0003817	0.0016774
85389	small nucleolar RNA, C/D box 14C	snoRNA	SNORD14C	-1.56	3.66E-05	0.0001969
1.1E+08	small nucleolar RNA, C/D box 157	snoRNA	SNORD157	-1.53	0.0011762	0.0046476
26792	small nucleolar RNA, C/D box 57	snoRNA	SNORD57	-1.45	0.0016814	0.006414
692197	small nucleolar RNA, C/D box 77	snoRNA	SNORD77	-1.42	9.10E-05	0.0004542
1E+08	small nucleolar RNA, C/D box 12B	snoRNA	SNORD12B	-1.41	1.71E-10	1.96E-09
619498	small nucleolar RNA, C/D box 74	snoRNA	SNORD74	-1.4	3.57E-10	3.96E-09
692212	small nucleolar RNA, C/D box 99	snoRNA	SNORD99	-1.38	2.06E-07	1.61E-06
26783	small nucleolar RNA, H/ACA box 65	snoRNA	SNORA65	-1.36	3.48E-07	2.62E-06
9297	small nucleolar RNA, C/D box 29	snoRNA	SNORD29	-1.34	1.92E-05	0.0001088
692196	small nucleolar RNA, C/D box 76	snoRNA	SNORD76	-1.32	1.12E-07	9.06E-07
677798	small nucleolar RNA, H/ACA box 9	snoRNA	SNORA9	-1.29	3.65E-10	4.05E-09
1.1E+08	small nucleolar RNA, H/ACA box 22B	snoRNA	SNORA22B	-1.27	0.0028011	0.0101092
1.2E+08	small nucleolar RNA SNORA71	snoRNA	LOC124904974	-1.27	4.24E-05	0.0002254
1.1E+08	small nucleolar RNA, C/D box 19C	snoRNA	SNORD19C	-1.25	0.0018201	0.0068735
677836	small nucleolar RNA, H/ACA box 58	snoRNA	SNORA58	-1.25	0.0015119	0.0058299
594838	small nucleolar RNA, C/D box 100	snoRNA	SNORD100	-1.24	0.0009525	0.0038441
26776	small nucleolar RNA, H/ACA box 71B	snoRNA	SNORA71B	-1.23	4.82E-05	0.0002542
1E+08	small nucleolar RNA, C/D box 119	snoRNA	SNORD119	-1.23	0.0056359	0.0187861
116937	small nucleolar RNA, C/D box 83A	snoRNA	SNORD83A	-1.22	6.72E-05	0.0003449
9299	small nucleolar RNA, C/D box 30	snoRNA	SNORD30	-1.19	5.78E-05	0.0003007
1.1E+08	small nucleolar RNA, C/D box 134	snoRNA	SNORD134	-1.15	1.22E-05	7.11E-05
692204	small nucleolar RNA, C/D box 88C	snoRNA	SNORD88C	-1.14	0.0026196	0.0095339
692089	small nucleolar RNA, C/D box 19	snoRNA	SNORD19	-1.13	0.002994	0.0107162
26822	small nucleolar RNA, C/D box 14A	snoRNA	SNORD14A	-1.11	3.63E-05	0.0001954
6044	small nucleolar RNA, H/ACA box 62	snoRNA	SNORA62	-1.11	6.09E-05	0.0003152
26804	small nucleolar RNA, C/D box 45B	snoRNA	SNORD45B	-1.08	0.0006314	0.0026424
9301	small nucleolar RNA, C/D box 27	snoRNA	SNORD27	-1.07	0.0009012	0.0036602
692088	small nucleolar RNA, C/D box 50B	snoRNA	SNORD50B	-1.05	0.0008777	0.0035724
594839	small nucleolar RNA, H/ACA box 33	snoRNA	SNORA33	-1.03	2.48E-05	0.0001373
1E+08	tRNA-Asn (anticodon GTT) 2-4	tRNA	TRN-GTT2-4	-8.53	1.56E-10	1.80E-09
4572	tRNA-Gln	tRNA	TRNQ	-7.98	1.65E-15	3.06E-14
4553	tRNA-Ala	tRNA	TRNA	-7.93	3.17E-09	3.14E-08
1E+08	tRNA-His (anticodon GTG) 1-8	tRNA	TRH-GTG1-8	-7.92	3.68E-09	3.62E-08
1E+08	tRNA-Pro (anticodon CGG) 2-1	tRNA	TRP-CGG2-1	-7.81	5.93E-09	5.71E-08
4556	tRNA-Glu	tRNA	TRNE	-7.67	1.13E-08	1.05E-07
1E+08	tRNA-Arg (anticodon TCG) 2-1	tRNA	TRR-TCG2-1	-7.57	1.80E-08	1.62E-07
7208	tRNA-Leu (anticodon TAG) 1-1	tRNA	TRL-TAG1-1	-7.57	1.95E-08	1.75E-07
4578	tRNA-Trp	tRNA	TRNW	-7.01	4.27E-27	1.53E-25
1E+08	tRNA-Leu (anticodon CAG) 2-1	tRNA	TRL-CAG2-1	-7	3.09E-12	4.26E-11
4571	tRNA-Pro	tRNA	TRNP	-6.75	1.80E-11	2.29E-10
1E+08	tRNA-Leu (anticodon TAA) 4-1	tRNA	TRL-TAA4-1	-6.58	5.36E-11	6.51E-10
1E+08	tRNA-His (anticodon GTG) 1-7	tRNA	TRH-GTG1-7	-6.43	1.44E-10	1.67E-09
1E+08	tRNA-Tyr (anticodon GTA) 2-1	tRNA	TRY-GTA2-1	-6.33	3.42E-06	2.21E-05
1.1E+08	tRNA-Gly (CCC) 6-1	tRNA	TRG-CCC6-1	-6.23	4.84E-06	3.04E-05
1E+08	tRNA-Lys (anticodon TTT) 3-1	tRNA	TRK-TTT3-1	-6.02	7.12E-13	1.05E-11
7239	tRNA-Val (anticodon AAC) 1-4	tRNA	TRV-AAC1-4	-5.98	1.18E-05	6.93E-05

1E+08	tRNA-His (anticodon GTG) 1-5	tRNA	TRH-GTG1-5	-5.98	1.13E-12	1.62E-11
1E+08	tRNA-Ser (anticodon CGA) 1-1	tRNA	TRS-CGA1-1	-5.98	1.21E-05	7.10E-05
4574	tRNA-Ser	tRNA	TRNS1	-5.85	6.78E-09	6.47E-08
1E+08	tRNA-Ser (anticodon GCT) 6-1	tRNA	TRS-GCT6-1	-5.75	4.64E-07	3.43E-06
4555	tRNA-Asp	tRNA	TRND	-5.68	1.55E-11	1.99E-10
1E+08	tRNA-Val (anticodon TAC) 1-1	tRNA	TRV-TAC1-1	-5.51	1.73E-06	1.17E-05
7218	tRNA-Pro (anticodon AGG) 2-6	tRNA	TRP-AGG2-6	-5.47	1.93E-45	1.31E-43
1E+08	tRNA-iMet (anticodon CAT) 1-8	tRNA	TRX-CAT1-8	-5.46	6.12E-05	0.0003166
1E+08	tRNA-Ile (anticodon AAT) 5-3	tRNA	TRI-AAT5-3	-5.43	7.65E-05	0.0003876
1E+08	tRNA-Pro (anticodon TGG) 1-1	tRNA	TRP-TGG1-1	-5.36	1.11E-07	9.01E-07
4573	tRNA-Arg	tRNA	TRNR	-5.21	0.0001246	0.0006055
1E+08	tRNA-Cys (anticodon GCA) 8-1	tRNA	TRC-GCA8-1	-5.2	0.0001319	0.0006375
4564	tRNA-His	tRNA	TRNH	-5.2	5.07E-17	1.03E-15
1E+08	tRNA-Ile (anticodon AAT) 2-1	tRNA	TRI-AAT2-1	-5.16	1.38E-30	5.75E-29
1E+08	tRNA-Gln (anticodon CTG) 6-1	tRNA	TRQ-CTG6-1	-5.16	1.89E-14	3.21E-13
1E+08	tRNA-Ile (anticodon AAT) 8-1	tRNA	TRI-AAT8-1	-5.13	0.0001497	0.0007147
1E+08	tRNA-Thr (anticodon CGT) 4-1	tRNA	TRT-CGT4-1	-5.12	4.61E-62	4.67E-60
1E+08	tRNA-Val (anticodon CAC) 1-5	tRNA	TRV-CAC1-5	-5.09	6.08E-14	9.92E-13
1E+08	tRNA-Leu (anticodon TAG) 2-1	tRNA	TRL-TAG2-1	-4.99	8.16E-07	5.79E-06
1E+08	tRNA-Gly (anticodon GCC) 1-5	tRNA	TRG-GCC1-5	-4.81	1.76E-10	2.02E-09
1E+08	tRNA-Asn (anticodon GTT) 3-1	tRNA	TRN-GTT3-1	-4.76	2.61E-05	0.000144
1E+08	tRNA-Thr (anticodon AGT) 1-1	tRNA	TRT-AGT1-1	-4.66	4.64E-06	2.93E-05
4576	tRNA-Thr	tRNA	TRNT	-4.54	1.61E-57	1.51E-55
1E+08	tRNA-Tyr (anticodon GTA) 5-5	tRNA	TRY-GTA5-5	-4.47	1.04E-05	6.16E-05
1.1E+08	tRNA-Asn (anticodon GTT) 25-1	tRNA	TRN-GTT25-1	-4.46	1.08E-05	6.40E-05
1E+08	tRNA-Arg (anticodon CCT) 3-1	tRNA	TRR-CCT3-1	-4.4	5.69E-12	7.63E-11
1E+08	tRNA-Arg (anticodon TCT) 3-1	tRNA	TRR-TCT3-1	-4.33	0.0001073	0.0005278
7214	tRNA-Asn (anticodon GTT) 2-7	tRNA	TRN-GTT2-7	-4.29	0.0001247	0.0006058
1E+08	tRNA-Cys (anticodon GCA) 2-4	tRNA	TRC-GCA2-4	-4.19	0.0001945	0.0009095
4565	tRNA-Ile	tRNA	TRNI	-4.1	2.72E-24	8.29E-23
1E+08	tRNA-His (anticodon GTG) 1-9	tRNA	TRH-GTG1-9	-3.98	2.30E-07	1.78E-06
1E+08	tRNA-Gln (anticodon CTG) 5-1	tRNA	TRQ-CTG5-1	-3.97	2.59E-22	7.17E-21
1E+08	tRNA-Thr (anticodon AGT) 2-2	tRNA	TRT-AGT2-2	-3.95	0.0003569	0.0015768
1E+08	tRNA-Leu (anticodon CAG) 2-2	tRNA	TRL-CAG2-2	-3.91	0.0003728	0.0016417
1E+08	tRNA-Glu (anticodon TTC) 2-1	tRNA	TRE-TTC2-1	-3.88	0.0004438	0.0019241
23540	tRNA-Ser (anticodon TGA) 4-1	tRNA	TRS-TGA4-1	-3.82	0.0005079	0.0021694
4566	tRNA-Lys	tRNA	TRNK	-3.67	2.23E-07	1.73E-06
1E+08	tRNA-iMet (anticodon CAT) 1-1	tRNA	TRX-CAT1-1	-3.64	0.0007509	0.0030948
1E+08	tRNA-Leu (anticodon CAG) 1-6	tRNA	TRL-CAG1-6	-3.54	0.0010013	0.0040216
4511	tRNA-Cys	tRNA	TRNC	-3.54	7.70E-07	5.50E-06
4569	tRNA-Met	tRNA	TRNM	-3.47	1.78E-11	2.28E-10
1E+08	tRNA-Gly (anticodon TCC) 2-6	tRNA	TRG-TCC2-6	-3.37	7.50E-08	6.21E-07
1E+08	tRNA-Cys (anticodon GCA) 14-1	tRNA	TRC-GCA14-1	-3.3	0.0007043	0.0029201
1E+08	tRNA-Arg (anticodon TCG) 1-1	tRNA	TRR-TCG1-1	-3.23	0.0001524	0.0007263
1E+08	tRNA-Lys (anticodon CTT) 2-2	tRNA	TRK-CTT2-2	-3.21	3.40E-37	1.83E-35
1E+08	tRNA-Asn (anticodon GTT) 2-1	tRNA	TRN-GTT2-1	-3.08	4.25E-06	2.70E-05
1E+08	tRNA-Ile (anticodon AAT) 5-5	tRNA	TRI-AAT5-5	-2.98	0.0016437	0.006281
1E+08	tRNA-Ala (anticodon TGC) 4-1	tRNA	TRA-TGC4-1	-2.85	6.37E-05	0.0003284
1E+08	tRNA-Asp (anticodon GTC) 3-1	tRNA	TRD-GTC3-1	-2.65	0.003399	0.0120155
4568	tRNA-Leu	tRNA	TRNL2	-2.54	0.0018362	0.0069241
1E+08	tRNA-Lys (anticodon TTT) 5-1	tRNA	TRK-TTT5-1	-2.43	0.001072	0.0042701
1E+08	tRNA-Ser (anticodon AGA) 2-4	tRNA	TRS-AGA2-4	-2.14	0.0041716	0.0144166
1E+08	tRNA-Asp (anticodon GTC) 2-10	tRNA	TRD-GTC2-10	-1.72	0.001158	0.0045846
**Significantly upregulated transcripts regulated by *E. coli* strain K12**						
3604	TNF receptor superfamily member 9	protein-coding	TNFRSF9	7.02	2.05E-09	2.15E-08
6364	C-C motif chemokine ligand 20	protein-coding	CCL20	6.51	5.72E-85	9.05E-83
79094	ChaC glutathione specific gamma-glutamylcyclotransferase 1	protein-coding	CHAC1	6.39	0	0
54541	DNA damage inducible transcript 4	protein-coding	DDIT4	5.76	0	0
7127	TNF alpha induced protein 2	protein-coding	TNFAIP2	4.89	0	0
27189	interleukin 17C	protein-coding	IL17C	4.33	1.62E-06	1.17E-05
3383	intercellular adhesion molecule 1	protein-coding	ICAM1	4.14	0	0
2919	C-X-C motif chemokine ligand 1	protein-coding	CXCL1	4.06	0	0
5266	peptidase inhibitor 3	protein-coding	PI3	3.82	1.11E-22	3.20E-21
6367	C-C motif chemokine ligand 22	protein-coding	CCL22	3.72	1.04E-05	6.65E-05
3576	C-X-C motif chemokine ligand 8	protein-coding	CXCL8	3.62	0	0
54206	ERBB receptor feedback inhibitor 1	protein-coding	ERRFI1	3.6	0	0
79924	adrenomedullin 2	protein-coding	ADM2	3.56	7.62E-127	2.29E-124
1649	DNA damage inducible transcript 3	protein-coding	DDIT3	3.47	1.47E-286	1.25E-283
2921	C-X-C motif chemokine ligand 3	protein-coding	CXCL3	3.4	7.79E-132	2.44E-129
2920	C-X-C motif chemokine ligand 2	protein-coding	CXCL2	3.36	1.27E-61	1.25E-59
6376	C-X3-C motif chemokine ligand 1	protein-coding	CX3CL1	3.24	1.15E-16	2.34E-15
51129	angiopoietin like 4	protein-coding	ANGPTL4	3.24	2.01E-32	8.97E-31
93082	neuralized E3 ubiquitin protein ligase 3	protein-coding	NEURL3	3.22	2.38E-17	5.08E-16
84812	phospholipase C delta 4	protein-coding	PLCD4	3.18	5.62E-07	4.38E-06
5328	plasminogen activator, urokinase	protein-coding	PLAU	3.15	3.66E-35	1.76E-33
1435	colony stimulating factor 1	protein-coding	CSF1	3.1	7.14E-17	1.48E-15
9518	growth differentiation factor 15	protein-coding	GDF15	2.96	0	0
27113	BCL2 binding component 3	protein-coding	BBC3	2.91	1.02E-47	7.18E-46
330	baculoviral IAP repeat containing 3	protein-coding	BIRC3	2.89	0	0
57561	arrestin domain containing 3	protein-coding	ARRDC3	2.8	0	0
8632	dynein axonemal heavy chain 17	protein-coding	DNAH17	2.75	0.0002073	0.0010081
9235	interleukin 32	protein-coding	IL32	2.75	5.90E-24	1.79E-22

5743	prostaglandin-endoperoxide synthase 2	protein-coding	PTGS2	2.73	2.12E-210	1.30E-207
7128	TNF alpha induced protein 3	protein-coding	TNFAIP3	2.71	4.34E-116	1.13E-113
1437	colony stimulating factor 2	protein-coding	CSF2	2.67	8.93E-13	1.33E-11
6698	small proline rich protein 1A	protein-coding	SPRR1A	2.6	2.03E-11	2.64E-10
1491	cystathionine gamma-lyase	protein-coding	CTH	2.59	1.93E-65	2.00E-63
2069	epiregulin	protein-coding	EREG	2.56	5.05E-96	1.02E-93
57495	NACHT and WD repeat domain containing 2	protein-coding	NWD2	2.53	2.00E-07	1.66E-06
639	PR/SET domain 1	protein-coding	PRDM1	2.52	7.63E-09	7.57E-08
23657	solute carrier family 7 member 11	protein-coding	SLC7A11	2.4	0	0
9021	suppressor of cytokine signaling 3	protein-coding	SOCS3	2.39	1.34E-47	9.30E-46
12	serpin family A member 3	protein-coding	SERPINA3	2.38	2.69E-09	2.78E-08
80329	UL16 binding protein 1	protein-coding	ULBP1	2.33	1.68E-21	4.54E-20
57761	tribbles pseudokinase 3	protein-coding	TRIB3	2.28	4.13E-287	3.73E-284
7130	TNF alpha induced protein 6	protein-coding	TNFAIP6	2.27	0.0001423	0.0007116
7422	vascular endothelial growth factor A	protein-coding	VEGFA	2.22	0	0
3552	interleukin 1 alpha	protein-coding	IL1A	2.22	1.15E-05	7.28E-05
10397	N-myc downstream regulated 1	protein-coding	NDRG1	2.2	0	0
1850	dual specificity phosphatase 8	protein-coding	DUSP8	2.19	0.0008962	0.0038
133	adrenomedullin	protein-coding	ADM	2.16	4.81E-194	2.64E-191
4791	nuclear factor kappa B subunit 2	protein-coding	NFKB2	2.14	9.90E-282	7.60E-279
83667	sestrin 2	protein-coding	SESN2_1	2.13	0.0021387	0.0082624
1.2E+08	collagen alpha-1(III) chain-like	protein-coding	LOC124900217	2.13	1.91E-05	0.0001155
374	amphiregulin	protein-coding	AREG	2.13	7.02E-181	3.48E-178
23645	protein phosphatase 1 regulatory subunit 15A	protein-coding	PPP1R15A	2.12	1.41E-102	2.96E-100
2081	endoplasmic reticulum to nucleus signaling 1	protein-coding	ERN1	2.08	1.50E-136	5.01E-134
1.2E+08	uncharacterized LOC124900374	protein-coding	LOC124900374	2.06	1.59E-10	1.88E-09
4616	growth arrest and DNA damage inducible beta	protein-coding	GADD45B	2.06	1.47E-70	1.70E-68
1316	KLF transcription factor 6	protein-coding	KLF6	2.02	1.92E-233	1.28E-230
7436	very low density lipoprotein receptor	protein-coding	VLDLR	2.02	2.84E-24	8.78E-23
1847	dual specificity phosphatase 5	protein-coding	DUSP5	2.01	1.46E-112	3.62E-110
122953	Jun dimerization protein 2	protein-coding	JDP2	2	9.50E-91	1.76E-88
2113	ETS proto-oncogene 1, transcription factor	protein-coding	ETS1	1.95	4.40E-158	1.83E-155
9283	G protein-coupled receptor 37 like 1	protein-coding	GPR37L1	1.95	0.0007703	0.003311
467	activating transcription factor 3	protein-coding	ATF3	1.93	1.28E-54	1.11E-52
651746	ankyrin repeat domain 33B	protein-coding	ANKRD33B	1.91	8.49E-08	7.36E-07
9590	A-kinase anchoring protein 12	protein-coding	AKAP12	1.9	1.26E-17	2.73E-16
5366	phorbol-12-myristate-13-acetate-induced protein 1	protein-coding	PMAIP1	1.89	2.29E-88	4.04E-86
10019	SH2B adaptor protein 3	protein-coding	SH2B3	1.86	1.63E-44	1.03E-42
81788	NUAK family kinase 2	protein-coding	NUAK2	1.85	1.79E-14	3.07E-13
25841	ankyrin repeat and BTB domain containing 2	protein-coding	ABTB2	1.83	2.35E-254	1.64E-251
5293	phosphatidylinositol-4,5-bisphosphate 3-kinase catalytic subunit delta	protein-coding	PIK3CD	1.83	1.64E-14	2.82E-13
8553	basic helix-loop-helix family member e40	protein-coding	BHLHE40	1.81	4.11E-201	2.33E-198
1.2E+08	uncharacterized LOC124904396	protein-coding	LOC124904396	1.78	7.19E-10	7.99E-09
22822	pleckstrin homology like domain family A member 1	protein-coding	PHLDA1	1.77	1.36E-167	6.34E-165
135112	nuclear receptor coactivator 7	protein-coding	NCOA7	1.76	2.97E-88	5.18E-86
5209	6-phosphofructo-2-kinase/fructose-2,6-biphosphatase 3	protein-coding	PFKFB3	1.75	4.04E-282	3.26E-279
6385	syndecan 4	protein-coding	SDC4	1.7	0	0
1051	CCAAT enhancer binding protein beta	protein-coding	CEBPB	1.66	1.76E-86	2.95E-84
9586	cAMP responsive element binding protein 5	protein-coding	CREB5	1.65	8.53E-43	5.16E-41
5155	platelet derived growth factor subunit B	protein-coding	PDGFB	1.65	1.02E-264	7.45E-262
54436	SH3 domain and tetratricopeptide repeats 1	protein-coding	SH3TC1	1.65	4.90E-54	4.16E-52
1831	TSC22 domain family member 3	protein-coding	TSC22D3	1.64	8.02E-70	9.18E-68
1026	cyclin dependent kinase inhibitor 1A	protein-coding	CDKN1A	1.61	1.95E-84	3.05E-82
9709	homocysteine inducible ER protein with ubiquitin like domain 1	protein-coding	HERPUD1	1.61	5.59E-90	9.98E-88
27289	Rho family GTPase 1	protein-coding	RND1	1.6	3.91E-16	7.73E-15
27071	dual adaptor of phosphotyrosine and 3-phosphoinositides 1	protein-coding	DAPP1	1.6	2.02E-32	8.98E-31
602	BCL3 transcription coactivator	protein-coding	BCL3	1.58	2.00E-87	3.40E-85
1.2E+08	uncharacterized LOC122319436	protein-coding	LOC122319436	1.58	0.0022983	0.008817
1907	endothelin 2	protein-coding	EDN2	1.57	0.0003384	0.0015675
23046	kinesin family member 21B	protein-coding	KIF21B	1.57	6.86E-26	2.30E-24
10509	semaphorin 4B	protein-coding	SEMA4B	1.56	0	0
55359	serine/threonine/tyrosine kinase 1	protein-coding	STYK1	1.55	1.58E-22	4.51E-21
6509	solute carrier family 1 member 4	protein-coding	SLC1A4	1.54	3.57E-65	3.63E-63
440	asparagine synthetase (glutamine-hydrolyzing)	protein-coding	ASNS	1.54	2.36E-26	8.05E-25
84962	ajuba LIM protein	protein-coding	AJUBA	1.53	3.93E-128	1.21E-125
9308	CD83 molecule	protein-coding	CD83	1.51	3.13E-08	2.86E-07
353322	ankyrin repeat domain 37	protein-coding	ANKRD37	1.51	1.56E-38	8.28E-37
6536	solute carrier family 6 member 9	protein-coding	SLC6A9	1.51	2.09E-49	1.57E-47
5971	RELB proto-oncogene, NF-kB subunit	protein-coding	RELB	1.51	1.36E-94	2.69E-92
3460	interferon gamma receptor 2	protein-coding	IFNGR2	1.5	1.13E-20	2.91E-19
51554	atypical chemokine receptor 4	protein-coding	ACKR4	1.5	0.0024856	0.0094435
375033	platelet endothelial aggregation receptor 1	protein-coding	PEAR1	1.49	1.32E-14	2.30E-13
347902	adhesion molecule with Ig like domain 2	protein-coding	AMIGO2	1.49	1.60E-78	2.25E-76
5292	Pim-1 proto-oncogene, serine/threonine kinase	protein-coding	PIM1	1.49	1.22E-52	9.79E-51
4783	nuclear factor, interleukin 3 regulated	protein-coding	NFIL3	1.46	2.60E-85	4.20E-83
1839	heparin binding EGF like growth factor	protein-coding	HBEGF	1.45	1.73E-21	4.67E-20
3656	interleukin 1 receptor associated kinase 2	protein-coding	IRAK2	1.45	1.09E-66	1.16E-64
3556	interleukin 1 receptor accessory protein	protein-coding	IL1RAP	1.42	3.76E-61	3.65E-59
11221	dual specificity phosphatase 10	protein-coding	DUSP10	1.41	6.84E-14	1.11E-12
54407	solute carrier family 38 member 2	protein-coding	SLC38A2	1.4	0	0
54887	bridge-like lipid transfer protein family member 3A	protein-coding	UHRF1BP1	1.4	9.14E-159	4.01E-156
694	BTG anti-proliferation factor 1	protein-coding	BTG1	1.39	6.77E-94	1.31E-91

901	cyclin G2	protein-coding	CCNG2	1.39	5.26E-40	2.88E-38
80830	apolipoprotein L6	protein-coding	APOL6	1.39	1.95E-65	2.01E-63
112399	egl-9 family hypoxia inducible factor 3	protein-coding	EGLN3	1.39	3.62E-221	2.32E-218
9076	claudin 1	protein-coding	CLDN1	1.34	2.56E-121	7.01E-119
6699	small proline rich protein 1B	protein-coding	SPRR1B	1.33	6.52E-06	4.31E-05
23135	lysine demethylase 6B	protein-coding	KDM6B	1.33	3.85E-74	4.93E-72
2300	forkhead box L1	protein-coding	FOXL1	1.33	1.96E-09	2.06E-08
4792	NFKB inhibitor alpha	protein-coding	NFKBIA	1.32	1.14E-126	3.36E-124
1999	E74 like ETS transcription factor 3	protein-coding	ELF3	1.32	3.56E-142	1.27E-139
4084	MAX dimerization protein 1	protein-coding	MXD1	1.32	3.48E-77	4.64E-75
571	BTB domain and CNC homolog 1	protein-coding	BACH1	1.31	8.81E-187	4.51E-184
4739	neural precursor cell expressed, developmentally down-regulated 9	protein-coding	NEDD9	1.31	2.46E-82	3.78E-80
54676	GTP binding protein 2	protein-coding	GTPBP2	1.31	1.50E-164	6.75E-162
50486	G0/G1 switch 2	protein-coding	G0S2	1.31	1.49E-15	2.81E-14
3097	HIVEP zinc finger 2	protein-coding	HIVEP2	1.3	1.85E-47	1.28E-45
2669	GTP binding protein overexpressed in skeletal muscle	protein-coding	GEM	1.3	1.29E-12	1.89E-11
6274	S100 calcium binding protein A3	protein-coding	S100A3	1.3	4.17E-12	5.78E-11
23780	apolipoprotein L2	protein-coding	APOL2	1.29	1.48E-26	5.13E-25
114789	solute carrier family 25 member 25	protein-coding	SLC25A25	1.28	2.07E-137	7.07E-135
8876	vanin 1	protein-coding	VNN1	1.28	0.0038791	0.0140558
1942	ephrin A1	protein-coding	EFNA1	1.27	6.45E-108	1.44E-105
80853	lysine demethylase 7A	protein-coding	KDM7A	1.27	9.69E-67	1.04E-64
84327	zinc finger BED-type containing 3	protein-coding	ZBED3	1.27	2.77E-54	2.38E-52
3475	interferon related developmental regulator 1	protein-coding	IFRD1	1.27	1.43E-145	5.34E-143
222611	adhesion G protein-coupled receptor F2	protein-coding	ADGRF2	1.26	1.24E-12	1.82E-11
80149	zinc finger CCCH-type containing 12A	protein-coding	ZC3H12A	1.26	1.85E-81	2.79E-79
23764	MAF bZIP transcription factor F	protein-coding	MAFF	1.26	1.87E-68	2.12E-66
9819	TSC22 domain family member 2	protein-coding	TSC22D2	1.25	7.56E-73	9.21E-71
3038	hyaluronan synthase 3	protein-coding	HAS3	1.24	6.21E-58	5.74E-56
51175	tubulin epsilon 1	protein-coding	TUBE1	1.24	8.93E-38	4.63E-36
1735	iodothyronine deiodinase 3	protein-coding	DIO3	1.23	0.0017428	0.0068501
677	ZFP36 ring finger protein like 1	protein-coding	ZFP36L1	1.21	8.02E-122	2.24E-119
1820	AT-rich interaction domain 3A	protein-coding	ARID3A	1.21	1.51E-18	3.52E-17
8140	solute carrier family 7 member 5	protein-coding	SLC7A5	1.21	5.14E-202	3.03E-199
286343	leucine rich adaptor protein 1 like	protein-coding	LURAP1L	1.21	1.50E-30	6.15E-29
3909	laminin subunit alpha 3	protein-coding	LAMA3	1.2	1.86E-158	7.95E-156
2274	four and a half LIM domains 2	protein-coding	FHL2	1.2	1.89E-74	2.43E-72
1958	early growth response 1	protein-coding	EGR1	1.2	1.76E-21	4.72E-20
5723	phosphoserine phosphatase	protein-coding	PSPH	1.2	3.04E-106	6.66E-104
389384	BCL2 interacting protein 5	protein-coding	BNIP5	1.19	7.09E-05	0.0003833
65243	ZFP69 zinc finger protein B	protein-coding	ZFP69B	1.19	2.42E-27	8.72E-26
6461	SH2 domain containing adaptor protein B	protein-coding	SHB	1.19	2.81E-133	9.17E-131
3918	laminin subunit gamma 2	protein-coding	LAMC2	1.19	2.87E-21	7.58E-20
64332	NFKB inhibitor zeta	protein-coding	NFKBIZ	1.19	5.68E-66	5.97E-64
2353	Fos proto-oncogene, AP-1 transcription factor subunit	protein-coding	FOS	1.18	1.86E-53	1.51E-51
10620	AT-rich interaction domain 3B	protein-coding	ARID3B	1.17	1.10E-44	6.99E-43
23138	NEDD4 binding protein 3	protein-coding	N4BP3	1.17	4.03E-26	1.36E-24
1647	growth arrest and DNA damage inducible alpha	protein-coding	GADD45A	1.17	4.95E-25	1.60E-23
3455	interferon alpha and beta receptor subunit 2	protein-coding	IFNAR2	1.16	1.51E-35	7.29E-34
1106	chromodomain helicase DNA binding protein 2	protein-coding	CHD2	1.15	1.37E-108	3.14E-106
10125	RAS guanyl releasing protein 1	protein-coding	RASGRP1	1.13	1.37E-05	8.50E-05
9792	SERTA domain containing 2	protein-coding	SERTAD2	1.12	7.82E-79	1.11E-76
6520	solute carrier family 3 member 2	protein-coding	SLC3A2	1.12	5.57E-119	1.50E-116
23150	FERM domain containing 4B	protein-coding	FRMD4B	1.12	2.97E-18	6.78E-17
84283	transmembrane protein 79	protein-coding	TMEM79	1.12	8.01E-19	1.89E-17
80201	hexokinase domain containing 1	protein-coding	HKDC1	1.11	5.56E-49	4.14E-47
6776	signal transducer and activator of transcription 5A	protein-coding	STAT5A	1.11	1.28E-14	2.23E-13
5467	peroxisome proliferator activated receptor delta	protein-coding	PPARD	1.11	6.88E-52	5.41E-50
83931	serine/threonine kinase 40	protein-coding	STK40	1.11	1.30E-117	3.43E-115
6452	SH3 domain binding protein 2	protein-coding	SH3BP2	1.11	2.36E-123	6.83E-121
134266	GrpE like 2, mitochondrial	protein-coding	GRPEL2	1.11	5.95E-84	9.23E-82
604	BCL6 transcription repressor	protein-coding	BCL6	1.1	3.79E-24	1.17E-22
7031	trefoil factor 1	protein-coding	TFF1	1.1	5.32E-33	2.40E-31
54498	spermine oxidase	protein-coding	SMOX	1.1	6.31E-95	1.26E-92
8542	apolipoprotein L1	protein-coding	APOL1	1.09	6.38E-12	8.67E-11
3976	LIF interleukin 6 family cytokine	protein-coding	LIF	1.09	2.69E-77	3.62E-75
3914	laminin subunit beta 3	protein-coding	LAMB3	1.09	3.11E-108	7.01E-106
5272	serpin family B member 9	protein-coding	SERPINB9	1.06	2.35E-30	9.61E-29
145781	GCOM1, MYZAP-POLR2M combined locus	protein-coding	GCOM1	1.06	6.10E-05	0.0003345
64651	cysteine and serine rich nuclear protein 1	protein-coding	CSRNP1	1.05	1.21E-24	3.83E-23
6926	T-box transcription factor 3	protein-coding	TBX3	1.05	1.51E-110	3.56E-108
833	cysteinyl-tRNA synthetase 1	protein-coding	CARS1_1	1.04	1.53E-15	2.89E-14
2523	fucosyltransferase 1 (H blood group)	protein-coding	FUT1	1.04	3.45E-17	7.32E-16
80271	inositol-trisphosphate 3-kinase C	protein-coding	ITPKC	1.03	2.20E-32	9.74E-31
56978	PR/SET domain 8	protein-coding	PRDM8	1.02	7.76E-07	5.88E-06
8795	TNF receptor superfamily member 10b	protein-coding	TNFRSF10B	1.02	2.45E-115	6.26E-113
4088	SMAD family member 3	protein-coding	SMAD3	1.01	8.60E-187	4.51E-184
9314	KLF transcription factor 4	protein-coding	KLF4	1	2.31E-156	9.35E-154
1.2E+08	uncharacterized LOC124902449	ncRNA	LOC124902449	4.79	3.12E-11	3.99E-10
693201	microRNA 616	ncRNA	MIR616	2.96	4.72E-05	0.0002645
1E+08	microRNA 3189	ncRNA	MIR3189	2.62	2.78E-08	2.56E-07
1.2E+08	uncharacterized LOC124903676	ncRNA	LOC124903676	2.36	4.41E-07	3.49E-06

1.1E+08	uncharacterized LOC105371159	ncRNA	LOC105371159	2.31	1.98E-10	2.32E-09
1E+08	lung cancer associated transcript 1	ncRNA	LUCAT1	2.3	1.79E-48	1.29E-46
1.1E+08	uncharacterized LOC105370672	ncRNA	LOC105370672	2.02	4.29E-11	5.35E-10
1E+08	MIR210 host gene	ncRNA	MIR210HG_1	1.98	1.37E-16	2.79E-15
285830	HLA-F antisense RNA 1	ncRNA	HLA-F-AS1_1	1.85	4.59E-15	8.28E-14
1.2E+08	uncharacterized LOC124903520	ncRNA	LOC124903520	1.81	0.0001135	0.0005847
1.1E+08	uncharacterized LOC105369299	ncRNA	LOC105369299	1.69	2.96E-05	0.0001722
1E+08	uncharacterized LOC100130460	ncRNA	CAND1.11	1.68	6.57E-05	0.0003572
1.2E+08	uncharacterized LOC124909470	ncRNA	LOC124909470	1.62	2.01E-06	1.44E-05
1.1E+08	uncharacterized LOC105369344	ncRNA	LOC105369344	1.59	2.48E-24	7.73E-23
1.2E+08	uncharacterized LOC124904012	ncRNA	LOC124904012	1.59	1.59E-05	9.78E-05
1.2E+08	ERRFI1 divergent transcript	ncRNA	ERRFI1-DT	1.49	0.0001305	0.0006606
1.1E+08	CZ1P-ASNS readthrough	ncRNA	CZ1P-ASNS	1.48	0.0039708	0.0143439
1E+08	microRNA 5087	ncRNA	MIR5087	1.47	4.87E-06	3.28E-05
1.1E+08	uncharacterized LOC105370789	ncRNA	LOC105370789	1.47	0.001645	0.006516
1.2E+08	uncharacterized LOC124901011	ncRNA	LOC124901011	1.44	0.0029049	0.0108695
1.2E+08	uncharacterized LOC124904469	ncRNA	LOC124904469	1.37	0.0002682	0.0012698
1.1E+08	uncharacterized LOC105371912	ncRNA	LOC105371912	1.31	0.0007749	0.0033297
1.2E+08	uncharacterized LOC124907970	ncRNA	LOC124907970	1.24	4.08E-07	3.25E-06
1E+08	ATXN1 antisense RNA 1	ncRNA	ATXN1-AS1	1.24	1.59E-21	4.31E-20
220930	ZEB1 antisense RNA 1	ncRNA	ZEB1-AS1	1.21	8.96E-08	7.72E-07
1E+08	HIF1A antisense RNA 2	ncRNA	HIF1A-AS2	1.19	0.001411	0.0056844
1.2E+08	uncharacterized LOC124904743	ncRNA	LOC124904743	1.14	0.0004695	0.002116
1.1E+08	uncharacterized LOC107984862	ncRNA	LOC107984862	1.09	0.0054235	0.0188169
1E+08	RARA antisense RNA 1	ncRNA	RARA-AS1	1.07	0.0001372	0.0006892
1E+08	CEBPB antisense RNA 1	ncRNA	CEBPB-AS1	1.07	9.51E-05	0.0005006
1.1E+08	uncharacterized LOC105372579	ncRNA	LOC105372579	1.07	2.17E-15	4.04E-14
1E+08	ODC1 divergent transcript	ncRNA	ODC1-DT	1.04	9.02E-06	5.81E-05
1E+08	microRNA 3648-2	ncRNA	MIR3648-2	1.03	0.0013263	0.0053705
284185	long intergenic non-protein coding RNA 482	ncRNA	LINC00482	1.03	8.37E-05	0.0004445
23642	small nucleolar RNA host gene 1	ncRNA	SNHG1	1.01	7.36E-122	2.09E-119
1E+08	RIPK2 divergent transcript	ncRNA	RIPK2-DT	1	3.91E-10	4.45E-09
1E+08	RNA, variant U1 small nuclear 8	snRNA	RNVU1-8	1.59	6.91E-08	6.07E-07
55370	protein phosphatase 4 regulatory subunit 1 like (pseudogene)	pseudogene	PPP4R1L	1.31	1.90E-08	1.78E-07
1E+08	clustered mitochondria homolog pseudogene 3	pseudogene	CLUHP3	1.1	1.37E-16	2.78E-15
**Significantly downregulated transcripts regulated by *E. coli* strain K12**						
3310	heat shock protein family A (Hsp70) member 6	protein-coding	HSPA6	-4.29	1.79E-61	1.75E-59
3304	heat shock protein family A (Hsp70) member 1B	protein-coding	HSPA1B_1	-2.46	1.75E-11	2.29E-10
3304	heat shock protein family A (Hsp70) member 1B	protein-coding	HSPA1B_2	-2.34	1.70E-08	1.60E-07
4541	NADH dehydrogenase subunit 6	protein-coding	ND6	-2.25	7.75E-149	2.97E-146
3304	heat shock protein family A (Hsp70) member 1B	protein-coding	HSPA1B_4	-2.1	1.29E-54	1.12E-52
1.1E+08	uncharacterized LOC105371267	protein-coding	LOC105371267	-1.93	2.81E-10	3.24E-09
374393	FAM111 trypsin like peptidase B	protein-coding	FAM111B	-1.84	1.79E-151	7.04E-149
9134	cyclin E2	protein-coding	CCNE2	-1.71	1.12E-80	1.67E-78
29119	catenin alpha 3	protein-coding	CTNNA3	-1.68	0.0010747	0.0044619
3303	heat shock protein family A (Hsp70) member 1A	protein-coding	HSPA1A_4	-1.61	0.0021686	0.0083675
150	adrenoceptor alpha 2A	protein-coding	ADRA2A	-1.59	1.12E-22	3.22E-21
389058	Sp5 transcription factor	protein-coding	SP5	-1.56	3.59E-11	4.53E-10
56977	storkhead box 2	protein-coding	STOX2	-1.55	0.0035117	0.0128826
10008	potassium voltage-gated channel subfamily E regulatory subunit 3	protein-coding	KCNE3	-1.47	1.26E-80	1.85E-78
195828	zinc finger protein 367	protein-coding	ZNF367	-1.43	3.79E-85	6.06E-83
5453	POU class 3 homeobox 1	protein-coding	POU3F1	-1.43	0.0007202	0.0031245
22809	activating transcription factor 5	protein-coding	ATF5	-1.42	2.92E-82	4.44E-80
27122	dickkopf WNT signaling pathway inhibitor 3	protein-coding	DKK3	-1.41	0.0027656	0.0104018
9982	fibroblast growth factor binding protein 1	protein-coding	FGFBP1	-1.33	4.70E-10	5.33E-09
84541	kelch repeat and BTB domain containing 8	protein-coding	KBTBD8	-1.29	2.74E-17	5.84E-16
390205	leucine rich repeat containing 10B	protein-coding	LRRC10B	-1.26	0.0003095	0.0014461
8368	H4 clustered histone 13	protein-coding	H4C13	-1.26	2.26E-36	1.13E-34
2049	EPH receptor B3	protein-coding	EPHB3	-1.26	1.84E-48	1.33E-46
55237	vertebrae development associated	protein-coding	VRTN	-1.23	0.000352	0.0016255
8322	frizzled class receptor 4	protein-coding	FZD4	-1.18	8.54E-11	1.04E-09
134548	sosondowah ankyrin repeat domain family member A	protein-coding	SOWAHA	-1.17	0.000274	0.0012927
2861	G protein-coupled receptor 37	protein-coding	GPR37	-1.15	4.37E-13	6.66E-12
7316	ubiquitin C	protein-coding	UBC	-1.14	1.70E-73	2.12E-71
8549	leucine rich repeat containing G protein-coupled receptor 5	protein-coding	LGR5	-1.13	6.62E-20	1.64E-18
993	cell division cycle 25A	protein-coding	CDC25A	-1.13	3.07E-60	2.95E-58
9531	BAG cochaperone 3	protein-coding	BAG3	-1.12	1.36E-70	1.58E-68
4535	NADH dehydrogenase subunit 1	protein-coding	ND1	-1.12	2.31E-12	3.29E-11
51053	geminin DNA replication inhibitor	protein-coding	GMNN	-1.09	7.15E-53	5.75E-51
63939	family with sequence similarity 217 member B	protein-coding	FAM217B	-1.08	1.32E-39	7.21E-38
7481	Wnt family member 11	protein-coding	WNT11	-1.07	1.62E-23	4.79E-22
115207	potassium channel tetramerization domain containing 12	protein-coding	KCTD12	-1.07	7.19E-42	4.19E-40
3312	heat shock protein family A (Hsp70) member 8	protein-coding	HSPA8	-1.04	1.61E-27	5.83E-26
118738	zinc finger protein 488	protein-coding	ZNF488	-1.03	3.31E-39	1.78E-37
3337	DnaJ heat shock protein family (Hsp40) member B1	protein-coding	DNAJB1	-1.02	1.80E-85	2.97E-83
3306	heat shock protein family A (Hsp70) member 2	protein-coding	HSPA2	-1.01	0.0010944	0.0045314
4998	origin recognition complex subunit 1	protein-coding	ORC1	-1.01	6.56E-28	2.42E-26
3222	homeobox C5	protein-coding	HOXC5	-1.01	2.20E-10	2.57E-09
84517	actin related protein T3	protein-coding	ACTRT3	-1	4.16E-05	0.0002353
1.2E+08	uncharacterized LOC124907850	ncRNA	LOC124907850	-1.57	1.46E-06	1.06E-05
1.1E+08	long intergenic non-protein coding RNA 2747	ncRNA	LINC02747	-1.49	3.12E-20	7.89E-19
1.1E+08	uncharacterized LOC105379280	ncRNA	LOC105379280	-1.3	1.63E-12	2.37E-11

1.2E+08	uncharacterized LOC124903536	ncRNA	LOC124903536	-1.29	1.90E-06	1.36E-05
1.1E+08	uncharacterized LOC107987100	ncRNA	LOC107987100	-1.25	5.45E-08	4.85E-07
1.1E+08	uncharacterized LOC107985684	ncRNA	LOC107985684	-1.06	4.99E-05	0.0002786
1.2E+08	uncharacterized LOC124902235	ncRNA	LOC124902235	-1.05	0.0006067	0.0026784
1E+08	LIPT2 antisense RNA 1	ncRNA	LIPT2-AS1	-1.02	1.12E-08	1.09E-07
1.2E+08	uncharacterized LOC124900987	ncRNA	LOC124900987	-1.01	3.09E-05	0.0001792
85391	small nucleolar RNA, C/D box 14E	snoRNA	SNORD14E	-1.84	5.90E-14	9.63E-13
85390	small nucleolar RNA, C/D box 14D	snoRNA	SNORD14D	-1.47	0.0005439	0.0024224
4572	tRNA-Gln	tRNA	TRNQ	-5.16	5.25E-30	2.11E-28
4565	tRNA-Ile	tRNA	TRNI	-4.44	1.21E-21	3.30E-20
4578	tRNA-Trp	tRNA	TRNW	-4.36	1.81E-46	1.21E-44
4553	tRNA-Ala	tRNA	TRNA	-4.36	1.11E-06	8.20E-06
4511	tRNA-Cys	tRNA	TRNC	-4.2	4.31E-06	2.93E-05
4569	tRNA-Met	tRNA	TRNM	-3.91	3.52E-14	5.87E-13
4574	tRNA-Ser	tRNA	TRNS1	-3.76	3.03E-08	2.78E-07
4555	tRNA-Asp	tRNA	TRND	-3.68	9.32E-11	1.13E-09
4564	tRNA-His	tRNA	TRNH	-3.24	3.92E-17	8.28E-16
4566	tRNA-Lys	tRNA	TRNK	-2.92	2.39E-06	1.69E-05
4576	tRNA-Thr	tRNA	TRNT	-2.8	2.22E-41	1.29E-39
1E+08	tRNA-Glu (anticodon CTC) 1-5	tRNA	TRE-CTC1-5	-1.95	0.0005192	0.0023227

References

1. Wang Y-L, Fang M, Wang X-M, Liu W-Y, Zheng Y-J, Wu X-B, et al. Proinflammatory effects and molecular mechanisms of interleukin-17 in intestinal epithelial cell line HT-29. WJG. 2014;20(47):17924.

2. Ho K-H, Chen P-H, Chou C-M, Shih C-M, Lee Y-T, Cheng C-H, et al. A key role of DNA damage-inducible transcript 4 (DDIT4) connects autophagy and GLUT3-mediated stemness to desensitize temozolomide efficacy in glioblastomas. Neurotherapeutics. 2020;17(3):1212-27.

3. Chen J, Liu F, Lee SA, Chen S, Zhou X, Ye P, et al. Detection of IL-18 and IL-1β protein and mRNA in human oral epithelial cells induced by *Campylobacter concisus* strains. BBRC. 2019;518(1):44-9.

4. Lee SA, Wang Y, Liu F, Riordan SM, Liu L, Zhang L. Escherichia coli K12 Upregulates Programmed Cell Death Ligand 1 (PD-L1) Expression in Gamma Interferon-Sensitized Intestinal Epithelial Cells via the NF-κB Pathway. Infect Immun. 2020;89(1):e00618-20.
